# Analyses of the Pathways Involved in Early- and Late-Phase Induction of IFN-Beta during *C*. *muridarum* Infection of Oviduct Epithelial Cells

**DOI:** 10.1371/journal.pone.0119235

**Published:** 2015-03-23

**Authors:** Sishun Hu, Kristen L. Hosey, Wilbert A. Derbigny

**Affiliations:** 1 Department of Microbiology and Immunology, Indiana University School of Medicine, Indianapolis, Indiana, United States of America; 2 College of Veterinary Medicine, Huazhong Agricultural University, Wuhan, People’s Republic of China; University of California Merced, UNITED STATES

## Abstract

We previously reported that the IFN-β secreted by *Chlamydia muridarum*-infected murine oviduct epithelial cells (OE cells) was mostly dependent on the TLR3 signaling pathway. To further characterize the mechanisms of IFN-β synthesis during *Chlamydia* infection of OE cells *in vitro*, we utilized specific inhibitory drugs to clarify the roles of IRF3 and NF-κB on both early- and late-phase *C*. *muridarum* infections. Our results showed that the pathways involved in the early-phase of IFN-β production were distinct from that in the late-phase of IFN-β production. Disruption of IRF3 activation using an inhibitor of TBK-1 at early-phase *Chlamydia* infection had a significant impact on the overall synthesis of IFN-β; however, disruption of IRF3 activation at late times during infection had no effect. Interestingly, inhibition of NF-κB early during *Chlamydia* infection also had a negative effect on IFN-β production; however, its impact was not significant. Our data show that the transcription factor IRF7 was induced late during *Chlamydia* infection, which is indicative of a positive feedback mechanism of IFN-β synthesis late during infection. In contrast, IRF7 appears to play little or no role in the early synthesis of IFN-β during *Chlamydia* infection. Finally, we demonstrate that antibiotics that target chlamydial DNA replication are much more effective at reducing IFN-β synthesis during infection versus antibiotics that target chlamydial transcription. These results provide evidence that early- and late-phase IFN-β production have distinct signaling pathways in *Chlamydia*-infected OE cells, and suggest that *Chlamydia* DNA replication might provide a link to the currently unknown chlamydial PAMP for TLR3.

## Background

Epithelial cells lining the genital tract are the major cell type productively infected with *Chlamydia* during genital tract infections. The acute host response to *Chlamydia* is primarily initiated and sustained by these infected epithelial cells, resulting in an array of innate-immune cytokines and chemokines with chemo-attractant and pro-inflammatory functions being secreted in the genital tract [1,2]. Consistent with that paradigm, we previously reported that cloned murine oviduct epithelial (OE) cell lines responded to C. *muridarum* infection by secreting a plethora of inflammatory cytokines and chemokines into the supernatants, and that the acute inflammatory cytokines such as IL-6 and GM-CSF were triggered in a TLR2-dependent manner [3,4]. We subsequently showed that the C. *muridarum*-infected OE cells secreted substantial amounts of interferon beta (IFN-β); however, its synthesis was largely MyD88-independent, requiring TRIF, IRF3, and TLR3 [5,6].

IFN-β is an immunomodulatory type-I interferon that plays an important role in the switch from innate to adaptive immunity [7]. *Chlamydia* induces IFN-β expression in a variety of cell types including macrophages, fibroblast, endothelial, and epithelial cells [8–13]. Our previous investigations into the specific role of IFN-β induced during *C*. *muridarum* infection of OE cells revealed that IFN-β modulates the transcription of several other cytokines and chemokines induced during *Chlamydia* infection, and that IFN-β can restrict *C*. *muridarum* replication in TLR3-deficient OE cells [14]. Our findings in OE cells corroborate the investigations of others that demonstrate an important role for epithelial cells in the *Chlamydia*-induced syntheses of innate-immune mediators into the genital tracts during infection, and that the *Chlamydia*-induced IFN-β is an important modulator of the immune response.

In the present study, we further examined the *Chlamydia*-induced synthesis of IFN-β in OE cells in an attempt to better understand the mechanism(s) by which IFN-β is synthesized in these cells during the course of infection. Our goal was to further clarify the roles of the important signaling mediates IRF3, IRF7, and NF-κB in *Chlamydia*-induced IFN-β synthesis at various times post-infection, in an effort to ascertain the relative contributions of their respective signaling pathways to the overall IFN-β response in OE cells. Finally, we examined the roles of bacterial DNA replication and bacterial gene transcription in the *Chlamydia*-induced IFN-β synthesis, as we seek to identify the currently unknown chlamydial pathogen-associated molecular pattern (PAMP) that binds to and stimulates TLR3.

## Materials and Methods

### Cell culture and *C*. *muridarum* infection

Derivation of the Bm1.11 cloned oviduct epithelial cell line has been described previously [4]. The cloned OE cell lines are grown at 37°C in a 5% CO_2_ humidified incubator and maintained in epithelial cell media: 1:1 DMEM:F12K (Sigma-Aldrich, St. Louis, MO), supplemented with 10% HyClone fetal bovine serum (Thermo Scientific, Rockford, IL), 2mM l-alanyl-l-glutamine (GlutaMAX I; Life Technologies/Invitrogen, Carlsbad, CA), 5 μg/ml of bovine insulin, and 12.5 ng/ml recombinant human fibroblast growth factor-7 (keratinocyte growth factor; Sigma-Aldrich, St. Louis, MO) as previously described [4,6]. The cells were seeded in 24-well tissue culture plates and used when they reached 70–90% confluence.

For all experiments, the cells were infected with either 1 IFU or 10 IFU per cell of *C*. *muridarum* Nigg in 24-well culture plates containing 500 μl of epithelial cell medium as described previously [5]. The plates were centrifuged at 1,200 rpm (200 × g) in a table-top centrifuge for 1 h, then incubated at 37°C in a 5% CO2 humidified incubator with changes of medium as described for each experiment. *Mycoplasma* free *C*. *muridarum* Nigg, previously known as *C*. *trachomatis* strain MoPn, was grown and titrated in McCoy cells (ATCC, Manassas, VA) as previously described [4,15]. The infection experiments and those requiring neutralizing antibody always included mock-infected controls. The mock-infection control cells received an equivalent volume of epithelial cell culture medium without *C*. *muridarum*, chemical inhibitor, or IFN-β neutralizing antibody. Inactivation of EBs was carried out by heating to 56°C for 30 min as previously described [16]. To ensure that treated EBs were completely inactivated, viability was tested on McCoy cells as described above. No recoverable IFU was found after incubation of inactivated EBs on McCoy cells for a period of 30 h (data not shown).

### Antimicrobial susceptibility testing

Antimicrobial susceptibility testing of *C*. *muridarum* in Bm1.11 OE cells to the antibiotics ofloxacin and rifampicin was carried out using similar methodology for the minimum inhibitory concentration (MIC) testing as previously described [17]. Briefly, Bm1.11 cells were grown to confluence in 48-well plates before being infected with 10 IFU/ cell of *C*. *muridarum* and centrifuged at 1,200 rpm (200 x g) as described above. At 2 h of post-infection, culture wells were supplemented with DMSO-dissolved antibiotic in various concentrations. The cells were allowed to incubate in the presence of the antibiotic at 37°C in a CO_2_ incubator until 18h PI. After 18 h incubation, the medium containing antibiotic was replaced with fresh antibiotic-free media and allowed to incubate for an additional 12 h in a 37°C CO_2_ incubator. At 30 h post-infection, cells were harvested and the cell pellets were stored at −80°C till use. *C*. *muridarum* titers were determined on fresh McCoy cell monolayers as previously described [18].

### Inhibitor treatments of *C*. *muridarum*-infected OE cells

OE cells were infected with *C*. *muridarum* and at various times post-infection, the media was either replaced and/or supplemented with either 0.01 μg/ml of rifampicin (MP Biomedicals, Santa Ana, CA), 0.1 μg/ml ofloxacin (Sigma-Aldrich, St. Louis, MO), 10 x IC50 (110 nM) of the TBK-1 inhibitor BX-795 (Sigma-Aldrich, St. Louis, MO), or 75 μM of the NF-κB inhibitor JSH-23 (Sigma-Aldrich, St. Louis, MO). All reagents were dissolved in DMSO. The cells were allowed to incubate in the presence of these inhibitors for various time intervals. Supernatants were subjected to analyses of the *Chlamydia*-induced IFN-β synthesis by IFN-β specific ELISA. The concentrations used for the inhibitors were determined empirically as the minimum amount that can be used to significantly inhibit the respective pathways without significantly affecting overall chlamydial replication.

### Preparation of *Chlamydia*-infection conditioned medium

Bm1.11 OE cells were grown to confluence in 48-well plates before being infected with 10 IFU/ cell of *C*. *muridarum* as described above. For 4–8h time-point: either sterile PBS or cycloheximide dissolved in PBS was added to the cell supernatants to achieve a 1μM cycloheximide concentration immediately after centrifugation (0h post-infection), and cells were incubated at 37°C in 5% CO_2_ as described above. The supernatants containing cycloheximide were removed at 4h post-infection, and replaced with fresh epithelial-cell medium. *Chlamydia*-infected OE cells were returned to the 37°C CO_2_ incubator until 8h post-infection when the conditioned supernatants were collected and filtered through 22μM filter (Nalgene; Rochester NY) before being added to fresh Bm1.11 monolayers. For 18–22h time-point: either sterile PBS or cycloheximide dissolved in PBS was added to the *Chlamydia*-infected OE cell supernatants achieve a 1μM concentration at 14h post-infection, the OE cells were returned to the 37°C tissue culture incubator, and the supernatants containing cycloheximide were removed at 18h post-infection and replaced with fresh epithelial cell media. Cells were returned to the 37°C tissue culture incubator until 22h post-infection, and conditioned supernatants were collected and filtered through 22uM filter before being added to fresh Bm1.11 monolayers. Conditioned supernatants were incubated with the uninfected Bm1.11 monolayers for 4 hours before cells were harvested for RT-qPCR analyses.

### IFN-β and IFNAR1 neutralization

For all experiments, OE cells were either mock-treated or infected with 10 IFU/ cell *C*. *muridarum* for at least 1 h before the culture medium was supplemented with either antibody. The neutralizing antibodies and isotype controls were diluted and stored according to manufacturers’ instructions. To neutralize IFN-β activity, cell supernatants were supplemented with either 0.1 or 1 μg/ml of rabbit anti-IFN-β polyclonal antibody (Thermo Scientific, Rockford, IL), or the corresponding IgG control antibody (Thermo Scientific, Rockford, IL). The manufacturer’s recommended range for neutralization is listed as 0.2 μg/ml-1.0 μg/ml for this product. In the experiments to ascertain the impact of neutralizing IFN-β function at specific time-points on the total amounts of *Chlamydia*-induced IFN-β at 24 hours post-infection, either anti-IFN-β or IgG control antibody was added to the supernatants of infected cells at the specified time-point, followed by gentle swirling of the 24-well plate before returning to the 37°C tissue-culture incubator for the remainder of the 24h infection. Identical control wells were included for each time-point that were also infected with *Chlamydia*; however, the supernatants were instead collected at the specified time, and the amount of IFN-β at that time-point was measured by ELISA (see below). The amount of IFN-β synthesized pre- and post-addition of antibody was calculated by subtracting the IFN-β secreted in the control experiments, from the total amounts secreted at 24h in the experiments when antibody was added. To examine the impact of neutralizing IFN-β function on the transcription rates of gene products of signaling components of the type-1 IFN signaling pathway, we added 1ug/ml of either anti-IFN-β or IgG control antibody, and the antibody was allowed to remain in the cultures until the specified time-point in which the cellular RNA was harvested and analyzed by RT-qPCR.

To block the interferon-alpha receptor 1 (IFNAR1) mediated autocrine-paracrine signaling of type-1 IFN, we used a neutralizing monoclonal antibody against mouse IFNAR1 and the corresponding mouse IgG1 isotype control that were purchased from Biolegend (San Diego, CA). For neutralization of IFNAR1, we used either 1 μg/ml of IFNAR1 neutralizing antibody or 1 μg/ml of the isotype control antibody. The antibody was added to the cell supernatants at either 1 hour post-infection, or 1 hour after being mock-infected with epithelial cell medium lacking viable *Chlamydia* (recombinant IFN-β experiments) as described previously in [19]. Recombinant murine IFN-β was purchased from R&D Systems (Minneapolis, MN). Recombinant murine IFN-β was reconstituted, stored, and used at 50 U/ml in epithelial medium as described [14]. The recombinant IFN-β was allowed to remain in the cultures until the cells were analyzed for transcription of either IFNα-2 or IFN-β by RT-qPCR. Untreated cells and sucrose-phosphate-glutamate (SPG) buffer-only treatment were used as uninfected and mock-infected controls, respectively.

### Gene expression analysis by real-time quantitative PCR

Cellular mRNA was purified from the mock and experimentally treated OE cells using the RNeasy mini kit (Qiagen, Valencia, CA), according to the manufacturer's instruction. During purification, all RNA samples were treated with RNase-free DNase I (Qiagen, Valencia, CA) to remove genomic-DNA contamination. The RNA was quantitated using a Nanodrop spectrophotometer (ND-1000; Thermo Scientific, Rockford, IL), and subjected to cDNA synthesis using the iSCript cDNA synthesis kit (Invitrogen; Grand Island, NY). Quantitative real-time PCR (qPCR) was performed using an ABI 7000 thermal cycler (Bio-Rad, Hercules, CA) following cDNA synthesis. Specific primers for the iScript RT-PCR were used with SYBR Green kit (Bio-Rad, Hercules, CA) according to the manufacturer's protocol. Optimized primer pairs were either designed by using the Primer 3 design tool [20], or created using previously published primer sequences for the specified gene. The specific primer pairs (Invitrogen, Carlsbad, CA) for targeting specific genes are listed in **Table 1**. Each gene’s mRNA quantification was normalized to the amount of the *β-actin* housekeeping gene. Relative expression and fold change was calculated using 2^−ΔΔCT^ method as previously described [21].

**Table 1:** Primers for RT-qPCR.

	Sense Primer	Antisense Primer
IFN-β	5ʹ˗*AAGAGTTACACTGCCTTTGCCATC*˗3ʹ	5ʹ˗*CACTGTCTGCTGGTGGAGTTCATC*˗3ʹ
IFNα-2	5ʹ˗*AGCAGATCCAGAAGGCTCAA*˗3ʹ	5ʹ˗*CATTCCAAGCAGCAGATGAA*˗3ʹ
IFNα-4	5ʹ˗*TTCTGCAATGACCTCCATCA*˗3ʹ	5ʹ˗*TATGTCCTCACAGCCAGCAG*˗3ʹ
STAT1	5ʹ˗*CGGAGTCGGAGGCCCTAAT*˗3ʹ	5ʹ˗*ACAGCAGGTGCTTCTTAATGAG*˗3ʹ
STAT2	5ʹ˗*TTTGGCTACCTGGATTGAAGAC*˗3ʹ	5ʹ˗*GGCTGAATTTTCGCAAGTTATGC*˗3ʹ
IRF3	5’-*TGGACGAGAGCCGAACGAGGTT*-3’	5’-*CGAACTCCCATTGTTCCTCAGC*-3’
IRF7	5ʹ˗*CAATTCAGGGGATCCAGTTG*˗3ʹ	5ʹ˗*AGCATTGCTGAGGCTCACTT*˗3ʹ
β-actin	5ʹ˗*GGCTGTATTCCCCTCCATCG*˗3ʹ	5ʹ˗*CCAGTTGGTAACAATGCCATGT*˗3ʹ
IFNAR1	5’-*ACACAGTTTCGTGTCAGAGCA*-3’	5’- *AACGGATCAACCTCATTCCAC*-3’
TLR1	5’-*GTGAATGCAGTTGGTGAAGAAC*-3’	5’-*GCTCATTGTGGGACAAATCCAA*-3’
TLR2	5’-*CTTGTTTCTGAGTGTAGGGGCT*-3’	5’-*CGAACCAGGAGGAAGATAAACT*-3’
TLR3	5′-*CCCGTTCCCAACTTTGTAGATG*-3′	5′-*TGCCAAATACTCCCTTTGTTGAA*-3′
TLR5	5’-*CAGTATCAGCTGATGAGACATGAG*-3’	5’-*GACAGTACCGCAATAGGGATGG*-3’
TLR6	5’-*TACGGAGCCTTGATTTCCATGT*-3’	5’-*TGGACCTCTGGTGAGTTCTGAT*-3’
IL-6	5’-*CTATACCACTTCACAAGTCGGAGG*-3’	5’-*TGCACAACTCTTTTCTCATTTCC3*–3’
GM-CSF	5’-*GCCATCAAAGAAGCCCTGAA*-3’	5’-*GCGGGTCTGCACACATGTTA*-3’
CCL4	5’-*CCCACTTCCTGCTGTTTCTC*-3’	5’-*GAGGAGGCCTCTCCTGAAGT*-3’
CCL5	5’-*ACTCCCTGCTGCTTTGCCTAC*-3’	5’-*GAGGTTCCTTCGAGTGACA*-3’
CXCL9	5’-*GAGTTCGAGGAACCCTAGTGATAAGGAA*-3’	5’-*AGGTTTGATCTCCGTTCTTCAGTGTAGC*-3’
CXCL10	5’-*CGATGACGGGCCAGTGAGAATG*-3’	5’-*TCAACACGTGGGCAGGATAGGCT*-3’
IL-1α	5’-*AGGAGAGCCGGGTGACAGTA*-3’	5’-*AACTCAGCCGTCTCTTCTTCAGA*-3’
TNFα	5’-*TGTGGCTTCGACCTCTACCTC*-3’	5’-*GCCGAGAAAGGCTGCTTG*-3’
Omp1	5’-*GCCGCTTTGAGTTCTGCTTCCTC*-3’	5’-*CCAAGTGGTGCAAGGATCGCA*-3’
16S rRNA	5’-*GCAAGTCGAACGGAGCAATT*-3’	5’-*ACCCTTCCGCCACTAAACA*-3’

### Transfection of poly-IC into Bm1.11 cells

Transfections of poly-IC (Sigma-Aldrich; St. Louis, MO) were done by complexing either 10, 25, or 50 μg/ml poly-IC to Lipofectamine 2000 reagent (Invitrogen; Grand Island, NY) in serum-free media as previously described [5]. After 30 min incubation at room temperature, the poly-IC complexes were added to Bm1.11 cells for 1 h. After 1 h, the transfection medium was removed and replaced with fresh epithelial cell media, or fresh epithelial media supplemented with either BX-795 or JSH-23. IFN-β secreted into the medium after 24 h post-transfection was measured by ELISA.

### RNA interference

Mouse Silencer small interfering RNA (siRNA) (catalog no. AM16708) and the negative control siRNA (catalog no. AM4611) were obtained from Thermo Scientific. Bm1.11 OE cells and RAW264.7 macrophages were transfected with siRNA using Lipofectamine 2000 according to the manufacturer’s instructions. At 24 h post-transfection, the cells were used for further experiments and the level of IRF7 gene expression was determined by RT-qPCR.

### Western blotting

Bm1.11 cells were plated in 24-well tissue culture plates and grown to confluency. Subsequently, these cells were either si-RNA treated, mock-infected, or C. *muridarum*-infected for 24 h. After removal of the cell supernatants, the cells were gently washed with PBS. Soluble proteins were then extracted with a lysis buffer containing 50 mM Tris-HCl (pH 7.4), 150 mM NaCl, 1% Triton X-100, 0.1% SDS purchased from Imgenex (San Diego, CA), and a mixture of protease inhibitor cocktail at 1 μl/ml (Sigma); while incubated on ice for 30 minutes. Cell lysates were clarified by centrifugation at 14,000 rpm (2400 × g) and proteins were quantified and separated by SDS-PAGE as previously described [5]. After proteins were transferred to nitrocellulose transfer membranes (Bio-Rad), the transfer membranes were blocked according to manufacturer’s protocol with a protein-free Tris-buffered saline (pH 7.4) containing 0.05% Tween-20 blocking buffer purchased from Thermo-scientific, Pierce (Rockland, IL). The proteins were stained by immunoblotting with either a 1:1000 dilution of a murine antibody against the C. *muridarum* major outer membrane protein (MOMP) [22], a 1:1000 dilution of anti-IRF7 antibody [EPR4718] (Abcam; Cambridge, MA), or 1:5000 dilution of anti-β-actin monoclonal antibody (clone AC-15; Sigma-Aldrich). Protein-antibody complexes were detected by secondary blotting with a 1:10,000 dilution of horseradish peroxidase-conjugated goat anti-mouse polyclonal antibody (Thermo scientific, Pierce). Proteins were visualized using the ECL western blotting substrate (Thermo scientific, Pierce) as described in the manufacturer’s protocol.

### ELISA measurement of IFN-β and IL-6

Peptidoglycan (PGN-EC) from *Escherichia coli* serotype O111:B4 (125 endotoxin units (EU)/mg), ODN1826, and ODN1826 (control) were purchased from InVivogen, (San Diego, CA) Bm1.1 OE cells and RAW 264.7 macrophages were either mock-treated, treated with peptidoglycan (PGN), transfected with poly-IC, or infected with 10 IFU/ cell *C*. *muridarum*. Cells were incubated in the presence or absence of specific chemical inhibitors to measure the impact of the inhibitors/ infection on cytokine synthesis starting at 1 or 2h post-treatment/ post-infection. Supernatants were harvested for measuring IFN-β and IL-6 using an in-house ELISA as previously described [3,5]. The lower range of the assay sensitivity was 10 pg/ml for the IFN-β ELISA, and 50 pg/ml for the IL-6 ELISA. All experiments were repeated at least three times, and significance was determined using the statistical analyses described below.

### Statistical Analyses

Statistical analysis was carried out using Student's *t*-test. The *p* values <0.05 were considered statistically significant (*p < 0.05; **p < 0.01). For RT-qPCR data, relative expression and fold change was calculated using 2^−ΔΔCT^ method as previously described [21].

## Results

### A factor secreted early into the supernatants during infection is important for optimal IFN-β synthesis in *C*. *muridarum*-infected OE cells

**Fig 1:**
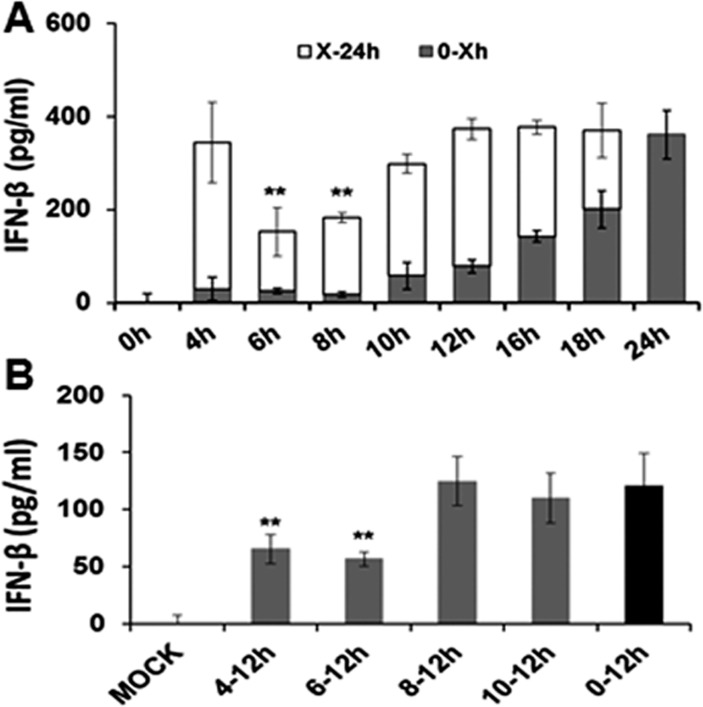
Kinetics of IFN-β production in *C*. *muridarum*-infected Bm1.1 OE cells. **(A)** Bm1.11 cells were infected with 10 IFU/ cell *C*. *muridarum* to measure the amount of IFN-β secreted into the supernatants for 24 hours. The supernatants were collected from the infected cells at each time point listed (denoted as 0-Xh), and replaced with fresh medium for the remainder of the 24 hour infection (denoted as X-24h). Total IFN-β secreted in the supernatants of the two phases was measured by ELISA. **(B)** Bm1.11 cells were infected with 10 IFU/cell *C*. *muridarum* to measure only the residual amount of IFN-β secreted into the supernatants after the media was replaced. Each time interval shows residual amounts of IFN-β synthesis up until 12hr post-infection. Horizontal bars represent mean ± SD of cytokine levels from experiments performed in triplicate. *Results are representative data from one of three independent experiments*. ** = *p< 0*.*01 compared to 24h C*. *muridarum infection control*.

**Fig 2:**
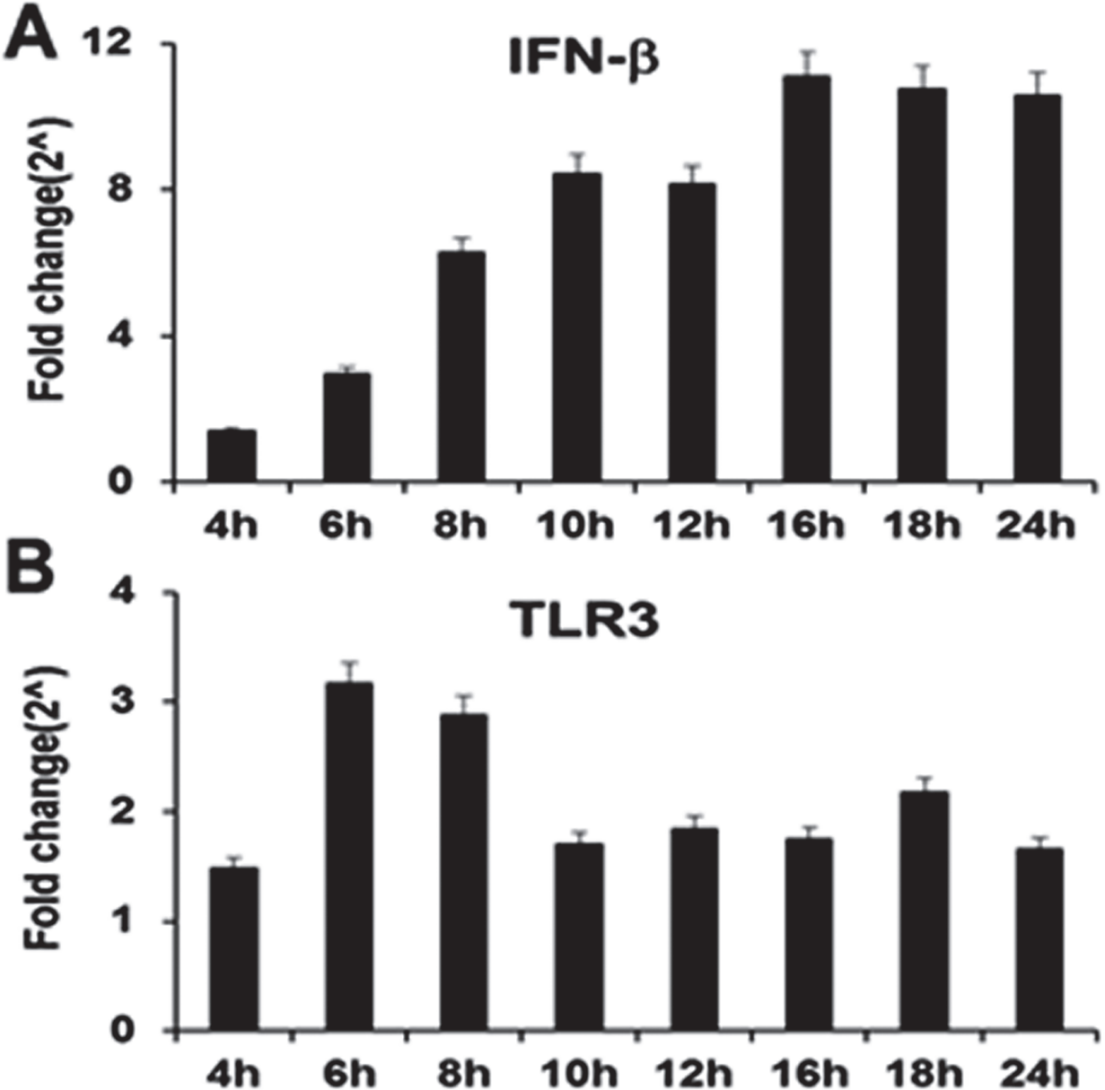
Gene expression levels of IFN-β and TLR3 during the course of *C*. *muridarum* infection. Bm1.11 cells were infected with 10 IFU/ cell *C*. *muridarum*, and the gene expression levels of: **(A)** IFN-β and **(B)** TLR3 were measured by RT-qPCR after total cell mRNA was harvested at each time-point indicated. *The results shown are representative of three independent experiments; Fold change is compared to Mock-infected controls*.

We previously reported that *C*. *muridarum* induced IFN-β synthesis in OE cells can be detected in the supernatants by IFN-β specific ELISA as early as 4 h post infection, and the amount of IFN-β secreted into the supernatant steadily increased throughout the course of infection, with highest concentration levels being observed between 18–20 h post-infection [5]. To ascertain the mechanism of *Chlamydia*-induced IFN-β production in OE cells, we examined the kinetics of IFN-β synthesis and the kinetics of the transcription of several known components of the IFN-β signaling pathways (Figs. 1 and 2). In order to first identify the time-point during the course of infection that was most critical for the optimal synthesis of IFN-β, Bm1.1 OE cells were infected with *C*. *muridarum* at 10 IFU /ml, the supernatants were harvested from the cells at various time points post-infection, and ELISA was used for measuring the amount of IFN-β synthesized up till that time (0-Xh). Fresh medium was added to the cells, and residual amount of IFN-β secreted into the supernatants up until 24 h PI was also measured by ELISA (Xh-24h).

As shown in Fig. 1A, IFN-β was detected in the supernatants as early as 4 h post-infection (4 h), and steadily increased in concentration up until 24 h PI, which corroborates the results of our earlier studies [5]. However, replacing the medium at either 6 h or 8 h post-infection appeared to have the greatest impact on the residual amount of IFN-β secreted into the supernatants of these cells (X-24h). Fig. 1B show results of a similar experiment examining the IFN-β synthesized within the first 12-hours of infection, whereby the media was replaced with fresh media at 4, 6, 8, or 10 h post-infection, and only the residual IFN-β synthesis (up until 12 h) was measured. As indicated, replacing the media at either 4 h or 6 h post-infection appeared to have the greatest impact on the residual amount of IFN-β secreted into the supernatants within the first 12 h-infection. Interestingly, replacing the media at 10 h post-infection had virtually no impact on the overall amount of residual IFN-β secreted into the supernatants within the first 12 h-infection with *Chlamydia*, suggesting that the cells are properly ‘conditioned’ for optimal *Chlamydia*-induced IFN-β within the first 10 h of infection. Our data imply that there is a secreted factor within the first 4–8 h post-infection that is important for the proper ‘conditioning’ of the OE cells for optimal *Chlamydia*-induced IFN-β synthesis.

To test our hypothesis that *Chlamydia* infection induces the synthesis of secreted factors that can acclimatize cells for the optimal expression of innate-immune factors such as IFN-β, we next examined the effect that *Chlamydia*-infection conditioned media has on the transcription rates of several gene products found in signaling pathways of innate-immune mediators in uninfected OE cells [3]. Supernatants were removed from *C*. *muridarum* infected OE cells for 4 hour intervals representing either early (4–8h PI) or late (18–22h PI) infection. The conditioned supernatants were filtered through 22μm filters and allowed incubate with uninfected Bm1.11 cells for 4hrs before monolayers were harvested for RNA isolation (See Materials and Methods). **S1 Fig.** shows RT-qPCR results of several genes found in the type-1 IFN signaling pathway (S1A Fig.), of various inflammatory mediators known to be induced during *Chlamydia* infection (S1B Fig.), and of the various TLRs known to be expressed and functional in the Bm1.11 OE cells [3] (S1C Fig.). As shown, most of the candidate genes were induced by the conditioned media removed from *Chlamydia*-infected OE cells representing both early- and late-times post-infection. Our data show that the majority of these genes were induced to higher levels when incubated with conditioned supernatants from late infection, with TLR3 and IFNα-2 being notable exceptions. OE cells that were infected with *C*. *muridarum* in the presence of the eukaryotic transcription inhibitor cycloheximide resulted in conditioned media that were able to induce expression of the candidate genes, but at substantially reduced levels. Collectively, these data support our hypothesis of a factor secreted into the supernatants of *Chlamydia* infected cells that can enhance the synthesis of innate immune modulators by OE cells *in vitro*. Results from OE cells infected in media containing cycloheximide suggest that these secreted factors are likely of cellular origin.

### IFN-β induced early during *Chlamydia* infection requires TLR3 signaling, but does not involve IFNAR

Our data suggest that there is a factor secreted early during *Chlamydia* infection that is important for optimal levels of IFN-β synthesis throughout the remaining course of infection. One possible explanation is that IFN-β itself is the secreted factor that induces its own expression in an autocrine-paracrine manner early during *Chlamydia* infection. We examined the kinetics of IFN-β gene induction to ascertain if there was a disproportionally higher induction of the IFN-β gene early during *Chlamydia* infection to support this theory. Fig. 2A shows the RT-qPCR results of the IFN-β gene throughout the 24 h course of *Chlamydia* infection of the Bm1.11 OE cells. As shown, the IFN-β gene expression was induced at 4 h post-infection, and the expression steadily increased until reaching its highest levels at 16 h. These results echo the findings of the IFN-β protein data (Fig. 1) that show that the IFN-β protein is secreted at the lowest levels early during infection, but that it was secreted at steadily increasing levels as the infection progresses.

We analyzed the gene-expression profile of TLR3 and as shown in Fig. 2B, TLR3 transcription was at its highest levels between 6 and 8 h post-infection, and its gene expression had diminished slightly between 10 and 16 h post-infection before trending upwards again at 18 h post-infection. Because TLR3 transcription levels are at its highest levels early during the course of *Chlamydia* infection, the TLR3 gene expression results suggest a more substantial role for TLR3 in the synthesis of IFN-β early during *Chlamydia* infection of OE cells as was previously reported [6,14]. TLR3 transcription appears to decrease later on during the infection, which is indicative that TLR3 likely plays a lesser role in the pathogenesis of *Chlamydia* infected OE cells at late times during the infection. These data corroborate our previous findings that suggest TLR3 plays a role in the early synthesis of IFN-β during *Chlamydia* infection of OE cells [14,19].

**Fig 3:**
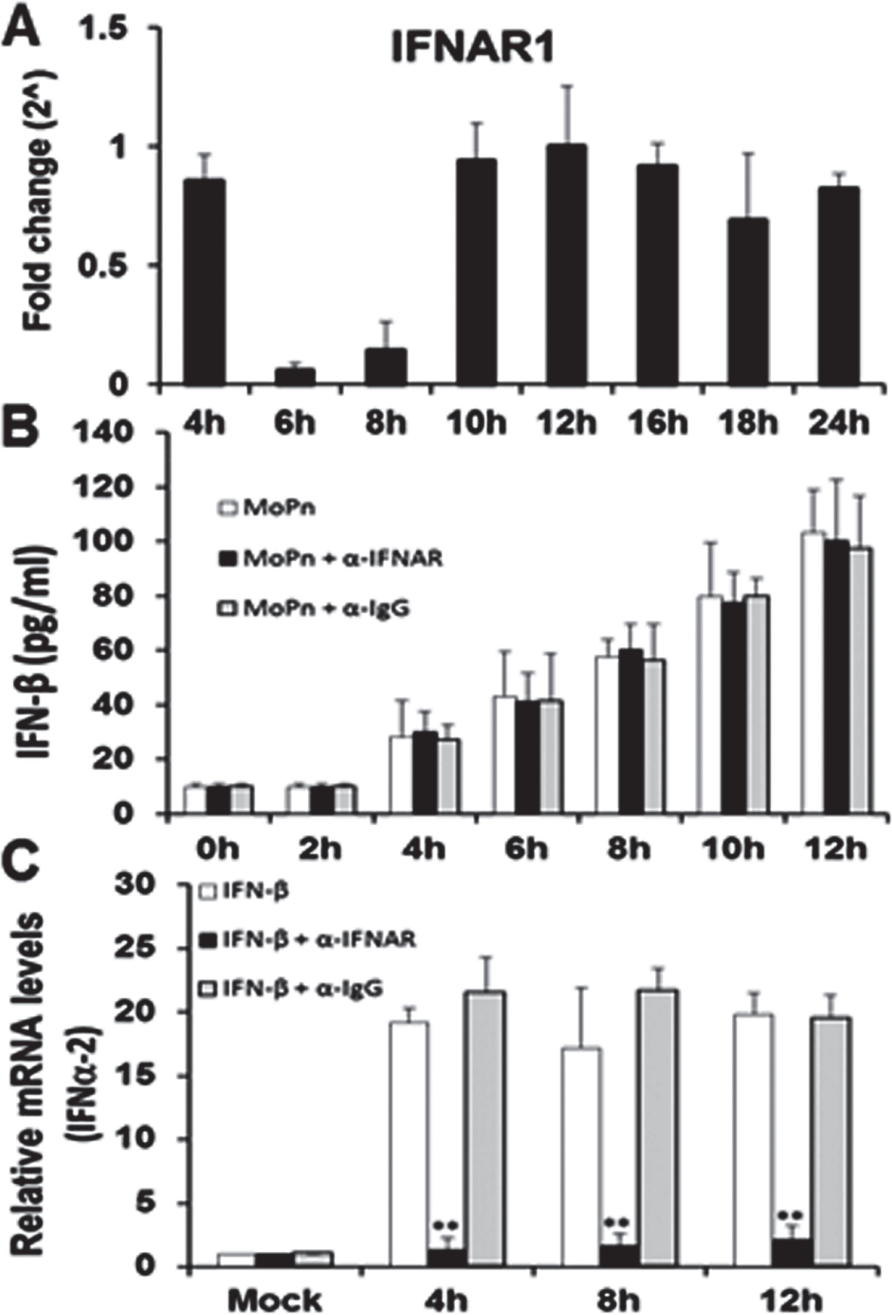
IFNAR1 early gene expression and function during the course of *C*. *muridarum* infection. **(A)** Bm1.11 cells were infected with 10 IFU/ cell *C*. *muridarum* and the transcription levels of IFNAR1 was measured by RT-qPCR after total cell mRNA was harvested at each time-point indicated. **(B)** Bm1.11 cells were infected with 10 IFU/cell *C*. *muridarum* in the presence or absence of either the IFNAR1-specific antibody (denoted as α-IFNAR) or the isotype control (denoted as α-IgG) at 1h post-infection, and the amount of IFN-β secreted into the supernatants during the first 12 h of infection was measured by ELISA. **(C)** Bm1.11 cells were incubated for 1h in media alone or in media containing either the IFNAR1-specific antibody or the isotype control, before adding 50U/ml recombinant murine IFN-β. Total cell mRNA was harvested after cells were exposed to recombinant IFN-β for an additional 12h, and IFNα-2 transcription was measured by RT-qPCR at the time-points listed. The results shown are representative of three independent experiments; Fold change and relative mRNA levels are compared to Mock-infected controls; ** = p< 0.*01 when compared to cells treated with recombinant IFN-β alone*.

We next examined the function and transcription profile of the interferon-alpha/beta receptor-1 (IFNAR1) in the Bm1.11 OE cells throughout the course of *Chlamydia* infection. As shown in Fig. 3A, IFNAR1 transcription remains consistent throughout the course of *Chlamydia* infection; however, there was a significant down-regulation in the transcription of IFNAR1 between 6 h and 8 h post-infection. The down-regulation of IFNAR1 transcription appears to require viable *Chlamydia* replication, since we were unable to noticeably impact IFNAR1 gene expression using heat-killed *C*. *muridarum* (**S2 Fig.**). The data reveal that the IFNAR1 receptor is at its lowest expression level during the 6–8 h time frame that we hypothesize a factor is secreted into the supernatants to promote the optimal synthesis of IFN-β during *Chlamydia* infection. Fig. 3B shows that blocking IFNAR1 signaling with IFNAR1-specific neutralizing antibody had no impact on the overall amounts of IFN-β secreted into supernatants of Bm1.11 OE cells during the first 12 h of *C*. *muridarum* infection. These data corroborate our transcription data (Fig. 3A), which suggest that IFNAR1 is likely not involved in the *Chlamydia*-induced synthesis of IFN-β between 6 h and 8 h post infection. Control experiments using uninfected Bm1.11 OE cells demonstrate that the IFNAR1-specific neutralizing antibody was effective in blocking the induction of the IFNα-2 gene transcription when uninfected OE cells were incubated in media supplemented with exogenous, recombinant murine IFN-β (Fig. 3C). As shown, the recombinant murine IFN-β substantially induced IFNα-2 transcription in these cells throughout the 12 h incubation period; however, the IFNα-2 gene induction was significantly reduced in the presence of the IFNAR1 neutralizing antibody. In contrast, IFNα-2 transcription was not affected by the presence of the IgG control antibody. Collectively, our data suggest that IFN-β is likely not the secreted factor between 6–8 h that is required for optimal *C*. *muridarum*-induced IFN-β synthesis in OE cells; however, the cycloheximide results of S1 Fig. suggest that it is likely a cell-derived factor. Additionally, we show that IFNAR1 is indeed functional in its role in the autocrine-paracrine induction of type-1 IFNs in the OE cells. However, our data suggest an unlikelihood that the unidentified factor secreted into the supernatants between 6–8 h post-infection will require the IFNAR1 receptor.

### *Chlamydia*-induced IFN-β is essential for optimal IFN-β synthesis late during infection of OE cells

**Fig 4:**
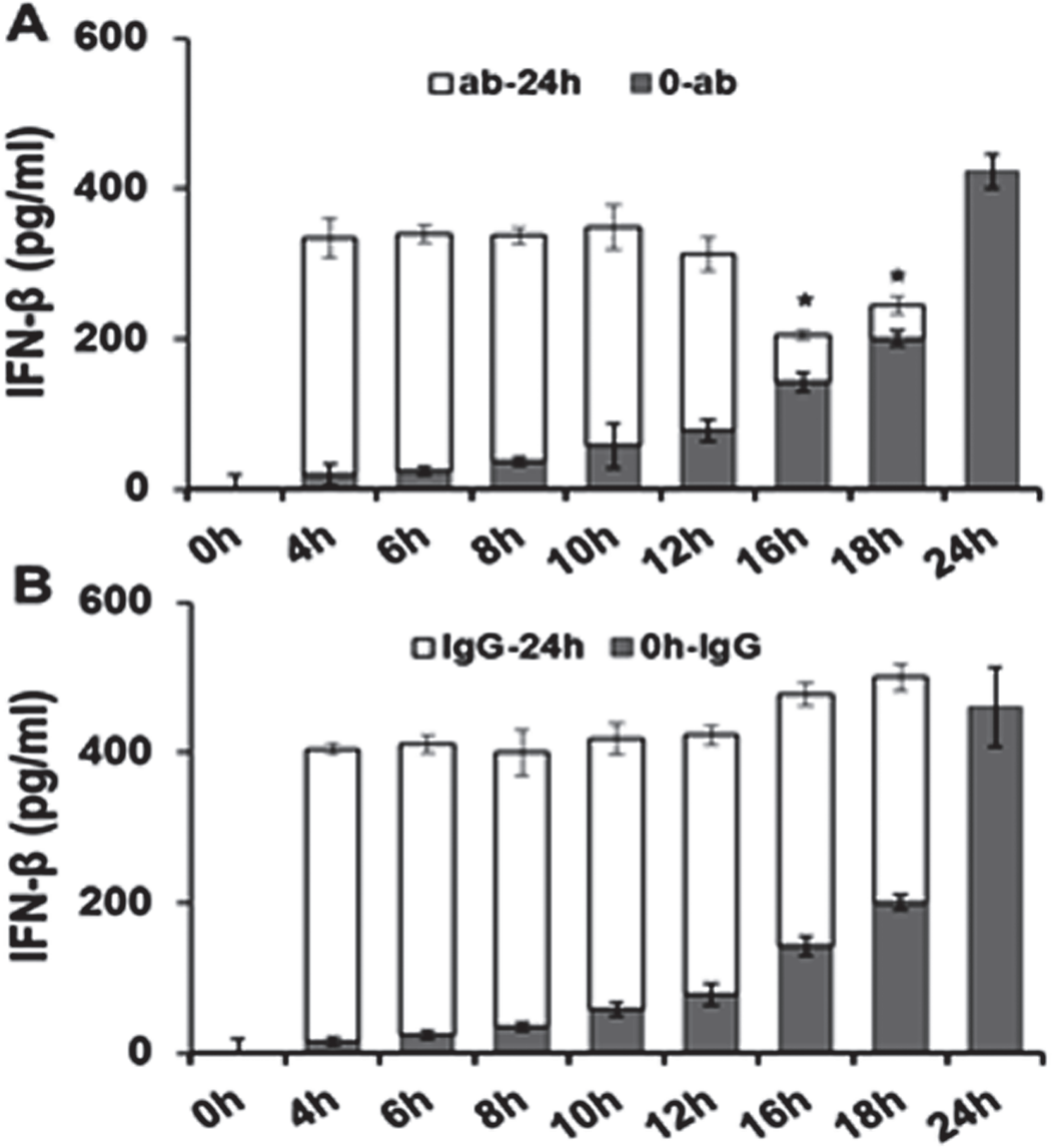
Disruption of IFN-β autocrine-paracrine pathways with neutralizing antibody. Bm1.11 cells were infected with 10 IFU/ cell *C*. *muridarum* to measure the amount of IFN-β secreted into the supernatants for 24 hours by ELISA. The supernatants were supplemented at each time-point listed with either 0.1μg/ml of: **(A)** IFN-β neutralizing antibody, or **(B)** isotype control antibody. The amount of IFN-β secreted into the supernatants after the addition of antibody (ab/IgG-24h) was estimated by subtracting out the amount of IFN-β secreted in control experiments in which supernatants were instead collected and assayed for IFN-β synthesis at the same time-point (see Materials and Methods). *Results are representative data from one of three independent experiments*. * = *p*< 0.05 *compared to 24h C*. *muridarum infection control*; *0-ab/IgG* *denotes “0hr PI until time antibody added”;* *ab/IgG-24h* *denotes “time antibody added until 24h PI”*.

To ascertain which phase of *C*. *muridarum* infection in OE cells is the most likely time in which IFN-β acts in an autocrine-paracrine manner to induce optimal levels of IFN-β, we examined the levels of IFN-β synthesis when neutralizing antibody to IFN-β was added at different intervals (Fig. 4). OE cells were infected with 10 IFU/cell *C*. *muridarum* and the medium was supplemented with either 0.1μg/ml IFN-β neutralizing antibody (Fig. 4A), or 0.1μg/ml IgG control antibody (Fig. 4B) at various times post-infection. The antibody amount used was titrated empirically using a customized version of the IFN-β neutralization assay [23], to a concentration that would neutralize only a portion of the total amount of IFN-β synthesized within 24 hours in *C*. *muridarum* infected OE cells. Results of the RT-qPCR based IFN-β neutralization assay revealed that 0.1μg/ml concentration of the IFN-β neutralizing antibody was sufficient to block 20–35% of IFN-β function (data not shown). Our goal was to measure the effectiveness of IFN-β neutralizing antibody in blocking the autocrine-paracrine induction of IFN-β during *Chlamydia* infection, and to ascertain which time-point during infection was the most important autocrine-paracrine induction. In this regard, we measured the total amount of IFN-β secreted into supernatants of *C*. *muridarum*-infected OE cells at 24h post-infection after the media was supplemented at various time-points with 0.1μg/ml concentration of either the IFN-β neutralizing antibody, or the corresponding IgG control.

As shown in Fig. 4A, the total amounts of IFN-β remain consistent until 16 h post-infection when IFN-β neutralizing antibody was added and the total amount of IFN-β secreted into the supernatants began to significantly decrease. Closer examination of the early-infection data indicated that the total amount of IFN-β secreted is not significantly changed by the addition of the neutralization antibody up through the 12 h post-infection. These data corroborate our previous hypothesis that the IFN-β synthesized early during infection plays little or no role augmenting the synthesis of itself, and that the mechanism for the early synthesis of IFN-β during *Chlamydia* infection likely does not involve autocrine-paracrine pathways. In contrast, neutralizing antibody added late during *C*. *muridarum* infection appeared to have a more profound impact on the synthesis of IFN-β late during infection. Because we were able to block IFN- β’s ability to induce its own synthesis at late times and not at early times, our data suggest a heavier reliance on the autocrine-paracrine mechanisms late. These findings support our hypothesis that these autocrine-paracrine pathways are likely most active after the 12 h time point.

### Late-stage IFN-β is synthesized via pathways that involve IRF7 in *Chlamydia*-infected OE cells

**Fig 5:**
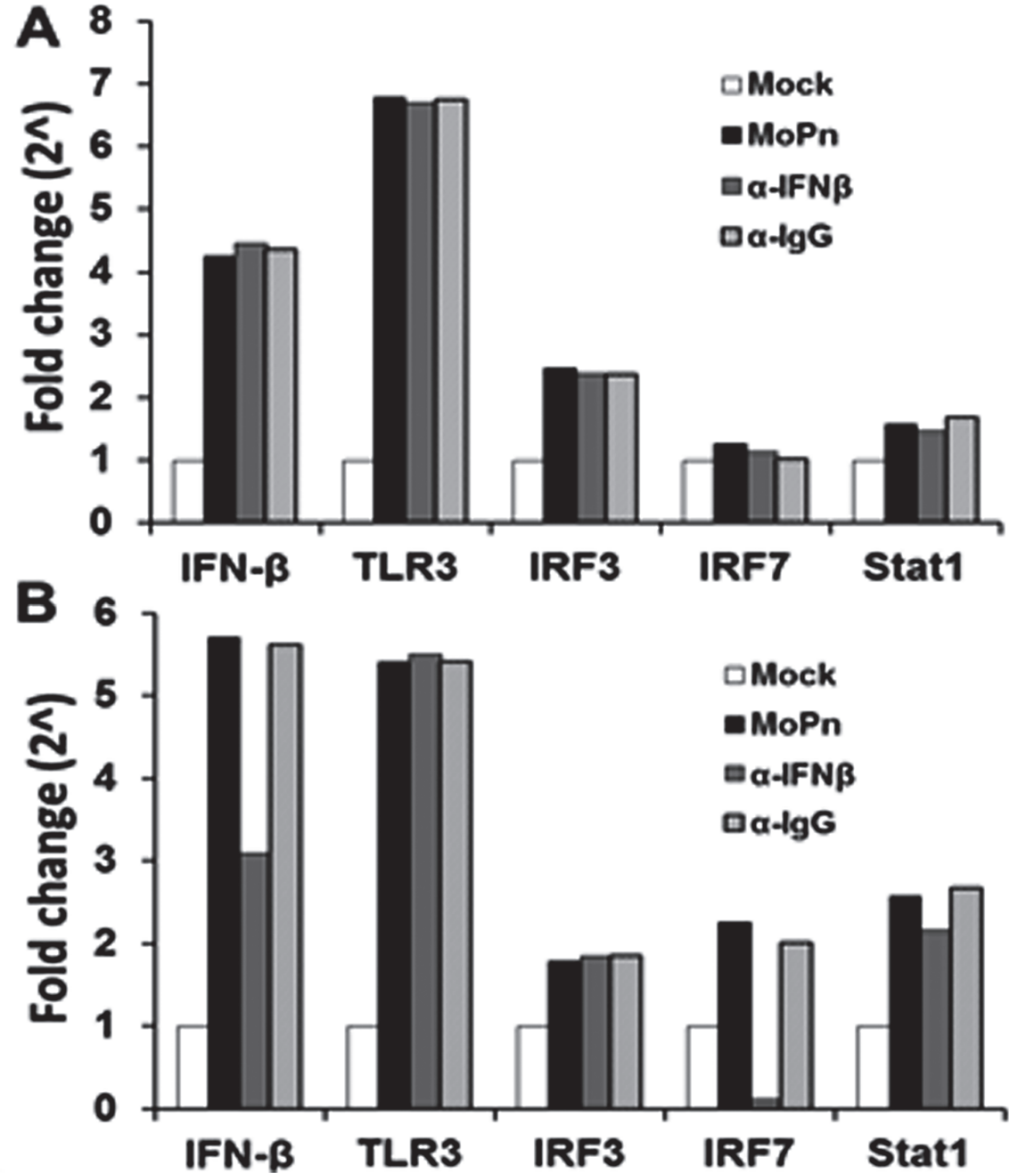
*C*. *muridarum*-induced IFN-β affects the gene expression levels of components found in the type-1 IFN signaling pathway. Bm1.1 cells were infected with 10 IFU/ cell *C*. *muridarum*, and the gene expression levels of IFN-β, TLR3, IRF3, IRF7, and Stat1 were measured by RT-qPCR after total cell mRNA was harvested at either early (12h) or late (24h) times post infection. **(A)** Transcription results of Bm1.11 OE cells harvested at 12h PI to measure the impact on transcription of candidate genes after cells were incubated from 4-to-12h PI in culture medium containing 1μg/ml of either IFN-β neutralizing antibody (α-IFNβ) or isotype control antibody (α-IgG). **(B)** Transcription results of these genes after cells were incubated in culture medium containing either antibody from 12-to-24h PI. *The results shown are representative of three independent experiments; Fold change is compared to Mock-infected controls*.

To further delineate the pathways involved in early vs late stage IFN-β synthesis, we examined the transcription levels of IFN-β, TLR3, IRF3, IRF7 and STAT1; which are proteins known to be required in the *Chlamydia*-induced synthesis of IFN- β [5,12,24–28]. As shown in Fig. 5A, OE cells were infected with *C*. *muridarum*, and the media was replaced with fresh media containing 2 μg/ml of either IFN-β neutralizing antibody or IgG control antibody at 4 h post-infection. The cells were harvested at 12 h and assessed for the transcription levels of the various genes involved in the *Chlamydia*-induced IFN-β synthesis pathways. As indicated, *C*. *muridarum* infection substantially induced the early transcription levels of IFN-β and TLR3; while Stat1 and IRF7 were not induced in any appreciable manner. Interestingly, we saw only a moderate induction of the IRF3 gene early during infection and the levels remained consistent throughout infection; however, there were no changes in the transcription of any of these genes after the addition of either IFN-β neutralizing antibody or IgG control.

We next assessed the transcription of these genes in cells in which the IFN-β neutralizing or IgG control antibody was added mid-to-late during *C*. *muridarum* infection of OE cells. The OE cells were allowed to incubate up until 12 h post-infection before the medium was replaced with fresh media containing 2 μg/ml of either the IFN-β neutralizing antibody or the IgG control antibody, and the cell were harvested at 24 h post-infection. As shown in Fig. 5B, all of these gene products were substantially induced late during *C*. *muridarum* infection; however, there was only a mild induction of the IRF3 gene, mimicking the early infection results (Fig. 5A). Addition of IFN-β neutralizing antibody had a dramatic impact in the down-regulation of IRF7 gene transcription, contrasting the early infection IRF7 results. We also saw diminished levels of IFN-β transcription when OE cells were incubated in the medium containing IFN-β neutralizing antibody when compared to *C*. *muridarum*-only reactions, suggesting a positive feedback loop for IFN-β by inducing IRF7 in OE cells. We saw no appreciable changes in the transcription rates of any of these genes early or late when media was replaced with fresh media containing the IgG control antibody.

We further examined the role of IRF7 in the IFN-β response to ascertain whether IRF7 protein had any direct role in *Chlamydia*-induced synthesis of IFN-β in OE cells (**S3 Fig.**). As shown in S3A Fig., we again showed that *C*. *muridarum* infection was able to induce transcription of IRF7 more substantially at late times during infection. As indicated, we were able to significantly reduce IRF7 mRNA using IRF7-specific si-RNA, which lead to diminished levels of total IRF7 protein and a significant decrease in IFN-β synthesis levels at late times during *Chlamydia* infection (S3B Fig. and S3C Fig.) Collectively, these data implicate a role for IRF7 in the late infection *Chlamydia*-induced synthesis of IFN-β in OE cells, and corroborates our previous finding that IFN-β plays a role in inducing itself late during *Chlamydia* infection.

### Inhibition of IRF3 but not NF-κB significantly impacts the early synthesis of IFN-β in *C*. *muridarum* infected OE cells

To confirm our hypothesis that IRF3 is involved in the early synthesis of IFN-β during *Chlamydia* infection of OE cells, we examined the impact of specific inhibitors of IRF3 and NF-κB on total IFN-β secretion. BX-795 is a potent and relatively specific inhibitor of IκB kinase ε (IKKε), phosphoinositide 3-dependent kinase 1 (PDK1), and TANK-binding kinase 1 (TBK1) [29,30]. BX-795 disrupts the function of TBK1 and IKKε, which then blocks the phosphorylation, nuclear translocation, and transcriptional activity of IRF3 [29]. JSH-23 is a potent inhibitor of NF-κB nuclear translocation, which does not affect the degradation of IκB kinases [31]. *C*. *muridarum*-infected Bm1.11 OE cells were allowed to incubate for various hours post-infection before adding either BX-795 (IRF3 inhibitor) or the NF-κB inhibitor JSH-23. Supernatants were harvested from the infected OE cells at 24h PI, and subjected to an ELISA assay to measure the total amounts of IFN-β secreted.

**Fig 6:**
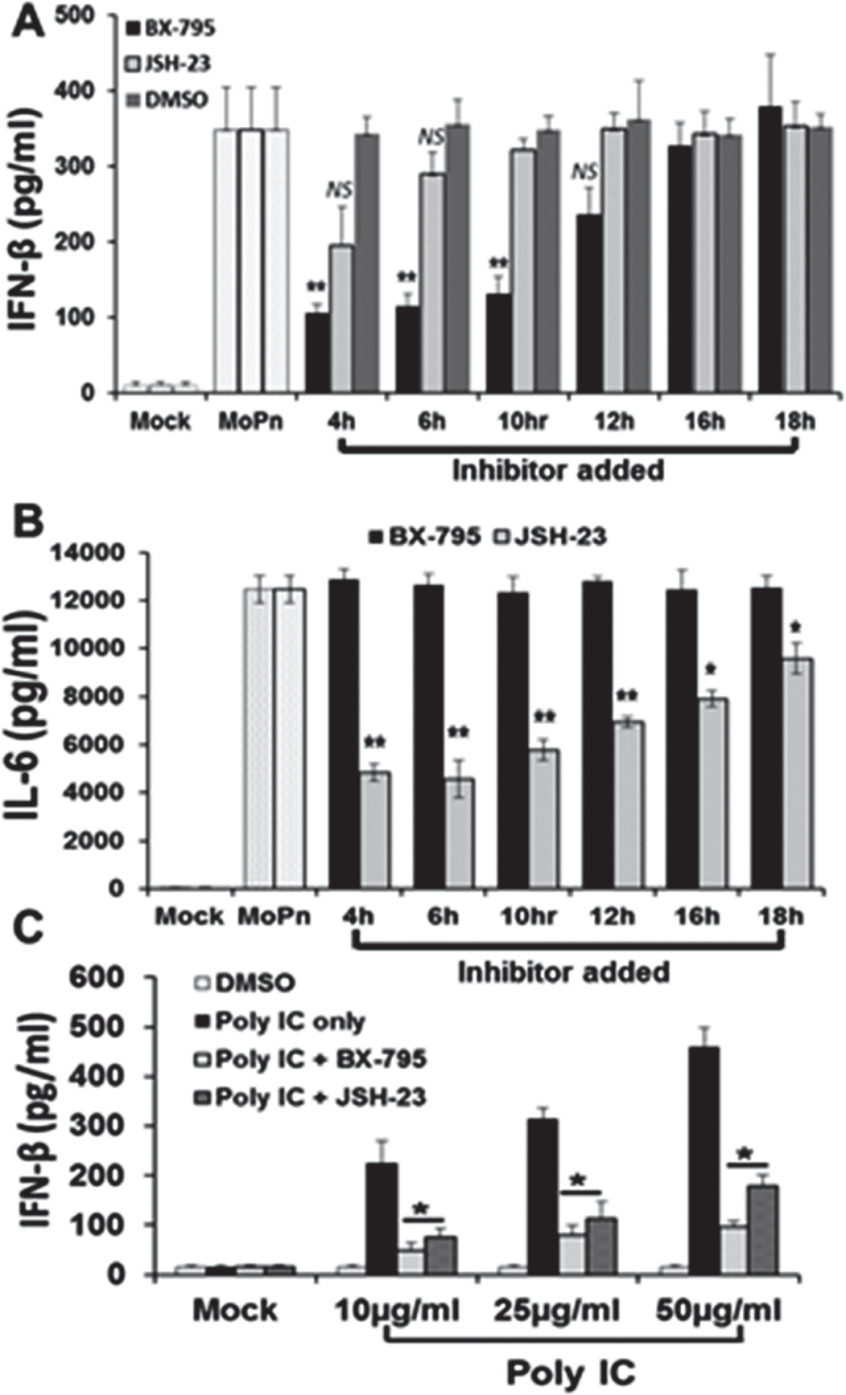
Inhibition of IRF3 and NF-κB affects early synthesis of IFN-β induced during *Chlamydia* infection. **(A)** Bm1.1 cells were infected with 10 IFU/ cell *C*. *muridarum* up until the indicated time-points when the media was supplemented with either the IRF3 inhibitor BX-795, the NF-κB inhibitor JSH-23, or the solubilizing agent DMSO as a negative control. Cells were allowed to incubate in the presence of each inhibitor from the time indicated until cell supernatants were harvested at 24h PI, and IFN-β secreted was measured by ELISA. **(B)** *C*. *muridarum*-infected Bm1.11 cells were allowed to incubate in the presence of each inhibitor from the time indicated until cell supernatants were harvested at 24h PI, and IL-6 secreted was measured by ELISA. **(C)** Uninfected Bm1.11 cells were either DMSO-treated, transfected with 10, 25, or 50 μg/ml poly-IC, or transfected with poly-IC prior adding the inhibitors BX-795 and JSH-23 to the cells 1h post-transfection. IFN-β secreted into the supernatants 24 h post transfection was measured by ELISA. *The results shown are representative of three independent experiments*. * = *p<0*.05; ** = p< 0.*01; NS = not statistically significant compared to poly-IC alone (C)*, *or 24h C*. *muridarum infection control without inhibitor (A and B; denoted as MoPn)*.

As shown in Fig. 6A, the addition of BX-795 resulted in significant decreases in the levels of IFN-β secretion when added between 4 and 10 h post-infection, while having little effect when the compound was added to the cells mid- and late-infection. Blocking NF-κB activity with JSH-23 seemed to have a minor effect on total IFN-β synthesis in *C*. *muridarum*-infected OE cells when added at 4 h post-infection; however, we routinely noticed only a minimal (but non-significant) decrease in IFN-β secreted when JSH-23 was added. In contrast, disrupting NF-κB activity with JSH-23 appeared to dramatically reduce the *Chlamydia*-induced synthesis of IL-6; however, IL-6 production was not affected by the addition of the IRF3 inhibitor BX-795 (Fig. 6B). We previously reported that IL-6 was induced via TLR2-dependent pathways in *C*. *muridarum*-infected OE cells [3], and our findings suggest a more critical role for NF-κB in the *Chlamydia*-induced synthesis of IL-6 throughout the course of infection. Positive control reactions (Fig. 6C) show that when added to the media at the identical inhibitory concentrations utilized in the *C*. *muridarum* infected OE cells, both BX-795 and JSH-23 were equally able to significantly reduce the amount of IFN-β secreted by Bm1.11 OE cells that were transfected with the TLR3 agonist poly-IC [32]. Negative control reactions using DMSO show no significant impact on the secretion of IFN-β during *Chlamydia* infection of OE cells (Fig. 6A), and we observed no significant effect on overall *C*. *muridarum* replication in the cells treated with either inhibitor or DMSO alone (data not shown).

Data from these experiments, in which we used BX-795 as a known inhibitor of PDK1, TBK1, and IKKε, showed significant reductions in early synthesis of IFN-β during *Chlamydia* infection. These findings suggest a disruption in IRF3 phosphorylation since TBK1 and IKKε are known to phosphorylate and regulate the function of IRF3 [29]. However, because IKKε and TBK1 also have some function in the phosphorylation and activation of IRF7 in human lung epithelial cells[33,34], it is possible that the disruption in *Chlamydia*-induced IFN-β synthesis is due to unwanted inhibition of IRF7 pathways by BX-795. IRF7 has been shown to be the major IRF functioning in type-1 IFN induced via TLR9 signaling [35,36]. Since Bm1.11 OE cells lack TLR9 and do not respond to the TLR9 agonist ODN 1826 [3], we used RAW 264.7 cells to specifically activate IRF7 via TLR9 signaling using ODN 1826. **S4A Fig.** shows that *C*. *muridarum* can induce IFN-β expression in the RAW264.7 cells, and that these cells respond to ODN1826 to secrete IFN-β in a TLR9-dependent manner. Transient disruption of IRF7 mRNA with si-RNA resulted in significant reductions in IFN-β synthesis, suggesting that ODN1826 signals through TLR9 via IRF7-dependent mechanisms (S4B Fig.). However, we were unable to significantly affect ODN 1826’s ability to induce IFN-β synthesis using up to 10x the concentration of BX-795 used in OE cell experiments (S4C Fig.). These data support our supposition that BX-795’s inhibitory effect on the *C*. *muridarum*-induced synthesis of IFN-β is likely due to disruption of IRF3 function in the early stages of the *Chlamydia*-induced IFN-β synthesis, and that IRF7 is likely not involved in the IFN-β synthesis early during infection.

### Transient inhibition of bacterial DNA replication decreases IFN-β secretion in *C*. *muridarum*-infected OE cells

We reported a major role for TLR3 in the *C*. *muridarum*-induced synthesis of IFN-β [6,14]. However, the exact chlamydial PAMP that binds to and stimulates TLR3 when the OE cells are infected with *C*. *muridarum* has not been identified. We hypothesized that either bacterial DNA or RNA were the likely PAMP(s) that binds to TLR3 during *Chlamydia* infection of OE cells. We utilized the bacterial DNA replication inhibitor ofloxacin [37], and the bacterial transcription inhibitor rifampicin [38] to ascertain which of these cell-cycle events had the greatest impact on *Chlamydia*-induced IFN-β synthesis in OE cells. Both ofloxacin and rifampicin are quite effective in the inhibition of overall chlamydial replication [39]. However, we sought to ascertain the optimal dose for these experiments whereby there was only minimal inhibition of the overall replication of *C*. *muridarum* in OE cells, even if there was a considerable decrease in either chlamydia DNA or RNA. Our goal was to ensure that decreases observed in IFN-β synthesis was due more to decrease in the respective nucleic acids, and less due to decreases in progeny totals. We infected Bm1.11 OE cells with 10 IFU/cell *C*. *muridarum* and, at 2 h post-infection, we replaced the medium with fresh medium supplemented with increasing concentrations of each antibiotic. The cells were allowed to incubate until 18 h post-infection before the supernatants containing antibiotic were removed and replaced with antibiotic-free fresh media. The cells were then harvested at 30 h post-infection and the *Chlamydia* titers were assessed using McCoy cell monolayers (See Materials and Methods).

**Fig 7:**
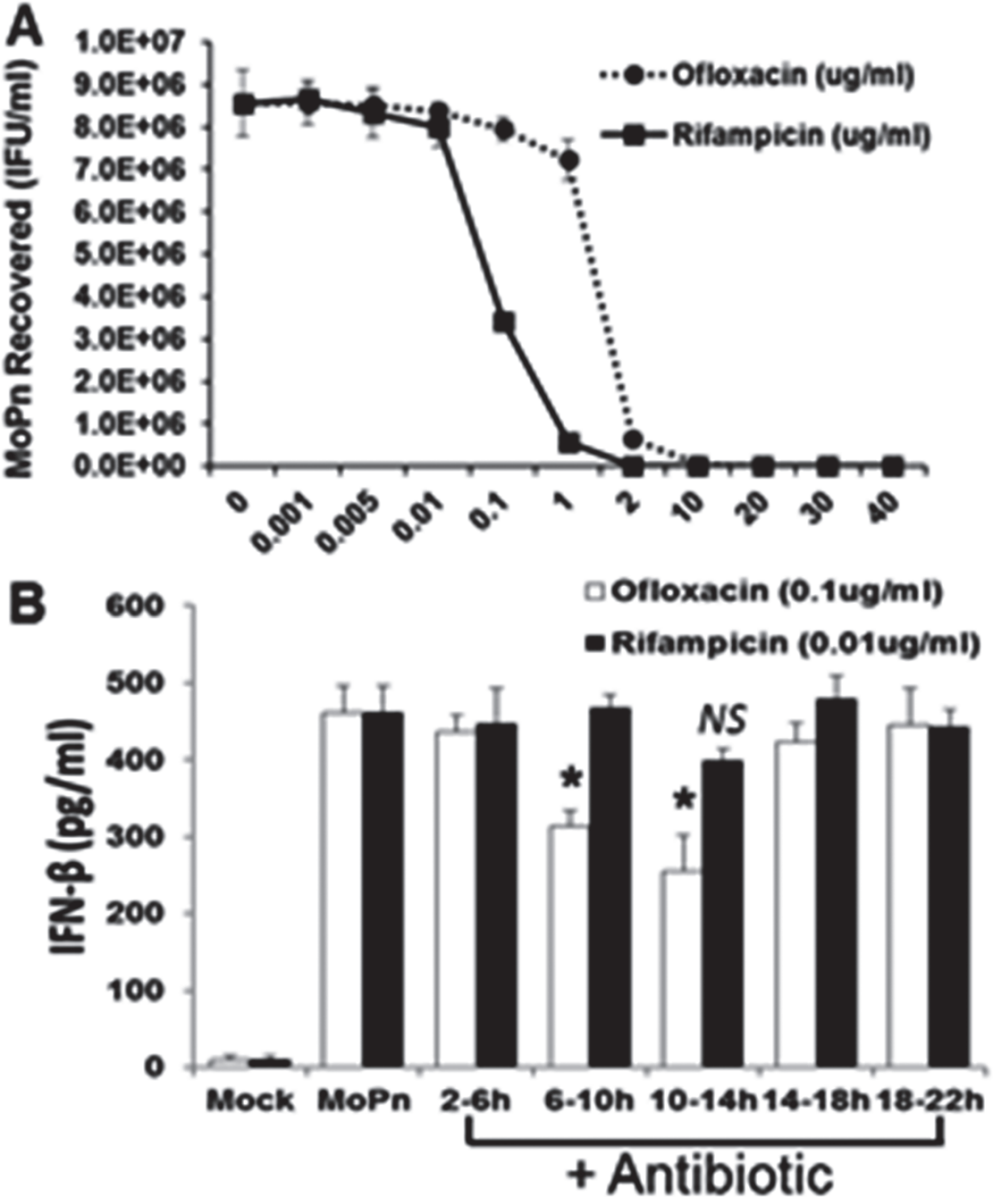
Role of bacterial DNA replication and bacterial transcription in *Chlamydia* induced IFN-β synthesis. **(A)** Bm1.11 cells were infected with 10 IFU/ cell *C*. *muridarum* and cells were incubated in the presence of increasing concentrations of either rifampicin or ofloxacin starting at 2h PI. The medium was replaced with antibiotic-free medium at 18h PI, cells were harvested at 30h PI, and *C*. *muridarum* titers (IFU/ml) were measured on McCoy cell monolayers as described in Materials and Methods. **(B)** Bm1.11 cells were infected with 10 IFU/ cell *C*. *muridarum* and cells were incubated in the presence of either 0.01 μg/ml rifampicin or 0.1 μg/ml ofloxacin for each 4h interval indicated, before cell supernatants were harvested at 24h PI and IFN-β secreted was measured by ELISA. *The results shown are representative of three independent experiments*. * = *p< 0*.*05; NS = not statistically significant compared to 24h C*. *muridarum infection control without antibiotic (denoted as MoPn)*.

As shown in Fig. 7A, rifampicin was a much more potent inhibitor of *Chlamydia* replication whereas we began to see a measurable decrease in *Chlamydia* replication starting at the 0.01 μg/ml concentration. We saw almost complete suppression of *C*. *muridarum* replication at the 2 μg/ml concentration or higher. In contrast, we did not see any significant reduction in *C*. *muridarum* replication until we reached the 1 μg/ml ofloxacin concentration. We did not see complete inhibition of *C*. *muridarum* replication until the 10 μg/ml concentration of ofloxacin. **S5 Fig.** demonstrates the corresponding reductions in chlamydial DNA and RNA when OE cells were infected in the presence of increasing doses of the respective antibiotic. We chose 0.01 μg/ml and 0.1 μg/ml concentrations of rifampicin and ofloxacin, respectively, as a suboptimal inhibitory dose for these experiments, whereby we begin to see only minor but equal reductions in chlamydial replication in these cells.

We infected Bm1.11 OE cells with 10 IFU/cell *C*. *muridarum* before removing (and saving) the supernatants, and replacing it with fresh media containing either 0.01 μg/ml rifampicin or 0.1 μg/ml ofloxacin at various time points of post-infection. The cells were incubated in the presence of antibiotic for 4 h intervals before the supernatants containing the antibiotic was removed, saved, and replaced with the original media that was removed prior to the addition of antibiotic. The cells were then allowed to incubate until 24 h post-infection, and the amount of IFN-β secreted into the combined supernatants (w and w/o antibiotic) was determined by ELISA. As shown in Fig. 7B, both antibiotics had varying impact on overall amounts of *Chlamydia*-induced IFN-β synthesis when the antibiotics were added between 2–18 h post-infection. However, we saw substantial and significant reductions in overall IFN-β levels when ofloxacin was added at the 6–10 h and the 10–14 h time points. We did not see a significant reduction in *Chlamydia*-induced IFN-β synthesis when rifampicin was added to the cells, though we did see a slight but non-significant decrease at the 10–14 h time point. There was no impact in the DMSO control reactions on *Chlamydia*-induced IFN-β synthesis (not shown).

**Fig 8:**
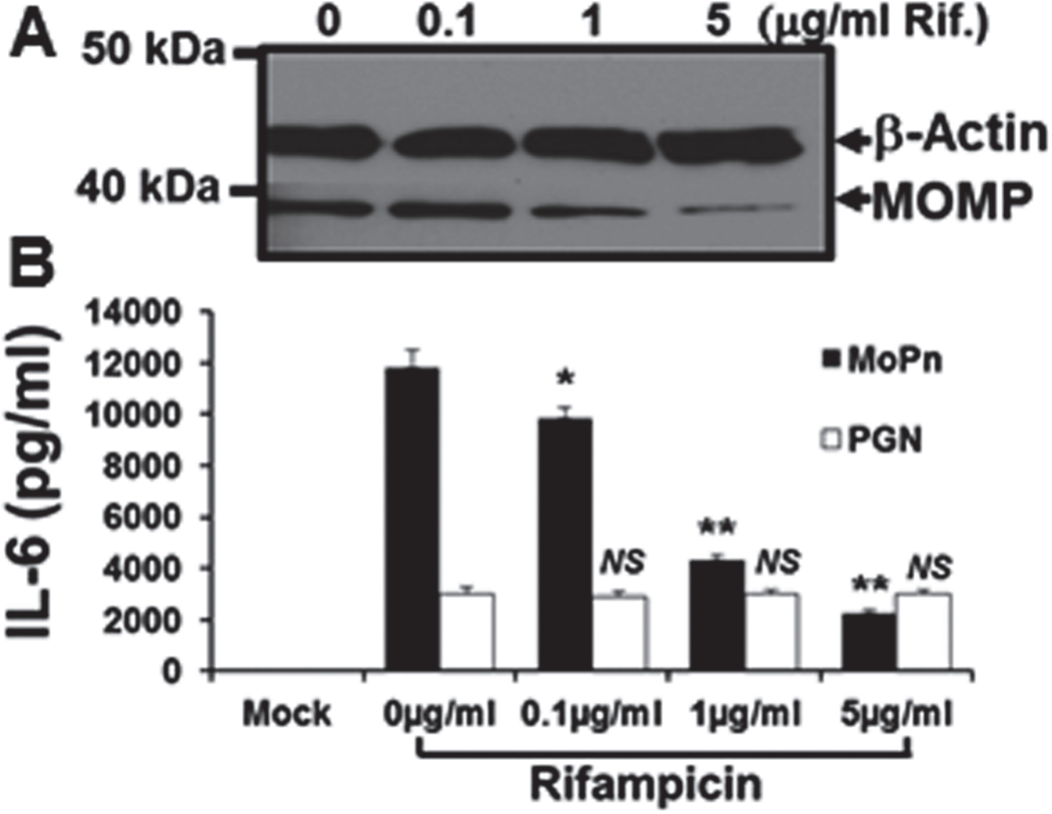
Rifampicin does not affect host-cell mechanisms. Bm1.11 cells were either mock-treated, treated with 10 μg/ml of the purified *E*. *coli* peptidoglycan (PGN), or infected with 10 IFU/ cell *C*. *muridarum*, and cells were incubated in the presence of increasing concentrations of rifampicin starting at 2h post-treatment/ infection. After 24 h post-treatment/ infection, cell lysates and supernatants were harvested for: **(A)** western-blot analyses for the *Chlamydia*-specific major outer membrane protein (MOMP) and cellular β-actin. **(B)** ELISA analyses of PGN-induced versus *Chlamydia*-infection specific cytokine expression. *The results shown are representative of three independent experiments*. * = *p<0*.05; ** = p< 0.*01; NS = not statistically significant compared to control cells without rifampicin (0* μ*g/ml); MoPn denotes Chlamydia infection*.

Fig. 8 shows control reactions addressing the unlikely (but possible) effects of rifampicin on eukaryotic transcription, and the non-specific immunomodulatory effects known to occur in rifampicin-treated eukaryotic cells [40]. As shown in Fig. 8A, *C*. *muridarum* infected OE cells expressed decreasing amounts of the chlamydial major outer membrane protein (MOMP) at 24 h post-infection, when incubated in medium supplemented with increasing amounts of rifampicin. However, despite utilizing rifampicin concentrations that correspond to 500X the concentration used in the *Chlamydia*-induced IFN-β experiments (*see Fig. 7B*); there was no equivalent reduction in the transcription of the host-cell structural protein β-actin. Fig. 8B shows that rifampicin had no immunomodulatory effect on the host-cell synthesis of IL-6 when uninfected Bm1.11 OE cells were induced for 24 h in medium supplemented with 10 μg/ml of the purified TLR2 agonist *E*. *coli* peptidoglycan (PGN) [41]. In contrast, there was a dose-dependent reduction in the amount of TLR2-dependent IL-6 [3] detected in the supernatants at 24 h in the *C*. *muridarum*-infected Bm1.11 OE cells. The reduction in the synthesis of IL-6 seen in the *Chlamydia*-infected OE cells (but not observed in the PGN treated uninfected OE cells), suggest that the diminished synthesis of IL-6 at high-dose concentrations of rifampicin are most likely due to substantially reduced transcription of the chlamydial TLR2 PAMPs during infection. Taken together, our data show that inhibition of chlamydial DNA replication has a much more dramatic impact on the *Chlamydia*-induced synthesis of IFN-β in OE cells than does the bacterial transcription. In effort to identify a putative chlamydial PAMP that triggers the TLR3-dependent IFN-β synthesis, our results are highly suggestive that the TLR3 PAMP in *C*. *muridarum*-infected OE cells is derived from products of *Chlamydia* DNA replication.

## Discussion

Our research focuses on the immunopathogenesis of *Chlamydia* infection and the overall contribution to the innate-immune response of epithelial cells lining the lumen of oviduct tissue. We have described the roles of TLR signaling in the synthesis of inflammatory cytokines, and understanding the mechanisms that lead to damage of the genital tract epithelium is paramount to development of therapeutic measures to prevent the sequelae of chronic genital tract *Chlamydia* infections. Using the OE cells as a model to study *in vitro Chlamydia* infections, we showed that *C*. *muridarum* induces IFN-β in a mostly TLR3-dependent manner, and we were the first to demonstrate TLR3-specific immune response to *Chlamydia* infection *in vitro* [6]. Our subsequent investigation into TLR3-dependent responses to *Chlamydia* infection in OE cells revealed that TLR3 stimulation resulted in the synthesis of a plethora of other inflammatory mediators, and that the TLR3-dependent IFN-β upregulated the gene expression of several of these innate-immune factors [14].

The mechanism by which *Chlamydia* induces IFN-β synthesis is an area of intense investigation. It has been shown that *Chlamydia* can induce IFN-β expression in a variety of cell types including macrophages, fibroblast, endothelial, and epithelial cells [8–13]. The *Chlamydia*-induced IFN-β synthesis in peritoneal macrophages was shown to be MYD88-dependent and likely signals through TLRs 7, 8, or 9 [11]. More recent results demonstrate that *Chlamydia*-induced IFN-β synthesis is independent of TLR signaling in multiple cell types, and strongly associated with the newly described stimulator of IFN gene (STING) protein [28,42]. Though the relationship between TLR3 signaling and STING has not been investigated in OE cells, it is a strong possibility that these pathways intersect during *Chlamydia* infection in these cells. The apparent differences observed in the pathways to IFN-β synthesis highlight the possibility that cell-type specific mechanisms of IFN-β synthesis are stimulated during *Chlamydia* infection. To this regard, we sought to ascertain the contribution of other signaling pathways contributing to the synthesis of IFN-β during *C*. *muridarum* infection of OE cells. In this report, we demonstrate that *Chlamydia*-induced IFN-β production in OE cells follows a biphasic pattern of expression, which involves the coordination of distinct signaling pathways for early and late infection synthesis of IFN-β that is similar to mechanisms described by others [28,43–47]. Our data proposes that the initial wave of IFN-β synthesized early during *Chlamydia* infection is TLR3-dependent and occurs through pathways involving IRF3; while late stage IFN-β synthesis is triggered by the type-1 IFN secreted into the supernatants, and signals through pathways that require IRF7.

We first showed that there was a factor secreted into the supernatants early during *C*. *muridarum* infection of OE cells infection that is critical for ‘conditioning’ the cells for the optimal induction of IFN-β. In order to identify this putative factor, we first examined whether IFN-β itself was this secreted factor since it is known that IFN-β can induce itself in an autocrine-paracrine manner [43,48]. However, our data indicate that IFN-β was not likely the secreted factor early during infection, though we have not ruled out the possibility of it having a role in inducing itself late during infection. Data from experiments in which OE cells were infected in the presence of cycloheximide show that inhibition of eukaryotic transcription has a negative impact on the synthesis of this putative secreted factor, and thus suggest this factor is derived from the OE cell machinery.

We showed that IFNAR1 was at its lowest expression levels between 6–8 h post-infection, and that inhibition of IFNAR1 mediated signaling with IFNAR1-specific neutralizing antibody had no effect on *Chlamydia*-induced synthesis of IFN-β during the first twelve hours post infection. In contrast, the IFNAR1-neutralizing antibody had a profound effect in disrupting the gene transcription of IFNα-2 when uninfected OE cells were induced with purified recombinant murine IFN-β. Our findings indicate that the IFNAR1 mediated pathway is indeed functional in OE cells, and that type-1 IFN can be induced through this pathway; however, data from these and our other ongoing investigation [19], show that IFNAR1 mediated signaling differentially induces the various type-1 IFNs throughout the course of *Chlamydia* infection in OE cells. The exact mechanism for the differential induction of type-1 IFNs through IFNAR1 signaling in OE cells is not known, though we hypothesize that it likely depends on the cytokine composition of the supernatants at that particular time during infection [49]. We surmise there must be some host cell or *Chlamydia*-induced component early during *Chlamydia* infection that either causes the preferential induction of IFNα through IFNAR1, or shuts off the ability of this pathway to induce IFN-β.

One possible candidate for the secreted factor that is critical for the optimal synthesis of IFN-β during Chlamydia infection of OE cells is cyclic-di-AMP (c-di-AMP). Cyclic-di-AMP was shown to be secreted by the intracellular bacteria *Listeria monocytogenes* and is a major contributor in inducing IFN-β [50]. In that study, it was shown that cyclic-di-AMP coordinates bacterial growth, cell wall stability, and the stress response; while also playing a crucial role in the establishment of bacterial infection. The specific cell-sensor for this cyclic dinucleotide in the IFN-β response has not yet been identified, but it was demonstrated that this pathway does function through downstream adaptor molecule STING. Subsequent to that study, it was demonstrated that *Chlamydia* synthesizes cyclic di-AMP, and that this metabolite was a prominent ligand for STING-mediated activation of type-1 IFN responses late during infection [42]. In that study, c-di-AMP was shown to be present in the cells of primary mouse lung fibroblasts and HEK293T late during *C*. *trachomatis* infections. It is not known whether the c-di-AMP can be detected in the culture medium during *Chlamydia* infection as in the *Listeria* studies, but because of its relationship to the endoplasmic reticulum (ER) membrane protein STING, it can be postulated that this di-nucleotide can be secreted from the cell through ER networks. Further studies are needed to ascertain whether c-di-AMP is synthesized during *C*. *muridarum* infection of OE cells, and whether its presence in the supernatants contribute to the overall levels of IFN-β synthesis during *Chlamydia* infection in OE cells.

We showed in IFN-β neutralization experiments that autocrine-paracrine synthesis of IFN-β in *C*. *muridarum*-infected cells occurs late during infection. In addition, we showed that this autocrine-paracrine induction occurs through pathways that likely signals through IRF7. Conversely, our data shows that IRF3 likely plays a larger role early during *Chlamydia* infection in OE cells. IRF3 and IRF7 are key activators of the transcriptional induction of IFNs α and β genes [36,47]. Both IRF3 and IRF7 play distinct and essential roles in the IFN-α/β response to virus infection [35,36]. In these and other studies, IRF3 was shown to be an important component of the immediate-early response to virus infection [51], while IRF7 is involved in the late induction phase of IFN expression during viral infection [45,46]. The importance of IRF3 and IRF7 in regulating the early and late phases of IFN expression during viral infection was demonstrated through the generation of IRF3 and IRF7 knock-out mice [47].

The requirement for activation of IRF3 and IRF7 in the IFN-β response to bacterial infection has been demonstrated in *Listeria* [52], *C*. *pneumonia* [53], and *Neisseria meningitidis* [54]. However, the exact temporal relationship between these IRFs has not been clearly defined in these studies. The roles for IRF3 and IRF7 have been examined in *C*. *muridarum* infection, and it was shown that there was an initial IRF3-dependent IFN-β secretion that forms a positive feedback loop by inducing IRF7; which is then required for maximal IFN-β expression during chlamydial infection of murine peritoneal macrophages [28]. Because the investigators in those studies demonstrated such a high dependence on STING during chlamydial induction of IFN-β, it was concluded that TLR signaling, MyD88, and TRIF were all dispensable for IFN-β upregulation during chlamydial infection of peritoneal macrophages.

Our data showed that IRF3 was involved early in the *Chlamydia*-induced synthesis of IFN-β, and that inhibiting IRF3 phosphorylation early with BX795 resulted in substantial reductions in overall IFN-β synthesis. Interestingly, we did not observe any effect on overall IFN-β synthesis when BX-795 was added late during *Chlamydia* infection, which suggest that IRF3 was not involved in the late-infection synthesis of IFN-β. BX-795 functions by inhibiting the auto-phosphorylation of IKKε, TBK1, and PDK1; and thereby preventing the nuclear translocation, phosphorylation, and transcriptional activity of IRF3 [29,30]. Although IKKε and TBK1 also have function in the phosphorylation and activation of IRF7 in human lung epithelial cells [34], there have been no reports detailing a disruption of IRF7 function in cells treated with BX-795, and we too were unable to disrupt IRF7-specific signaling pathways using this inhibitor in RAW264.7 cells (*see S4 Fig.*). The fact that BX-795 can quite effectively disrupt the function of IRF3, yet not have any impact on the function of IRF7, suggest that IKKε and TBK1 regulates these transcription factors via different mechanisms [33]. Because we were not able to disrupt late-infection synthesis of IFN-β in the *Chlamydia*-infected OE cells with BX-795, our results propose that the late-infection IFN-β synthesis involves the contributions from IRFs other than IRF3; which supports the observations of Pratner *et al*, demonstrating a role for IRF7 late during *Chlamydia* infection of peritoneal macrophages [28].

We investigated the putative role of IRF7 in the *Chlamydia*-induced IFN-β response in OE cells, and our data showed a substantial reduction in the transcription levels of IRF7, when media containing IFN-β neutralizing antibody was added to the cells late during *C*. *muridarum* infection. In contrast, there were no changes in the gene expression levels of IRF7 when IFN-β neutralizing antibody was added to the cells early during infection; which is indicative of IRF7 playing little to no role in the synthesis of IFN-β early during *C*. *muridarum* infection. These results were corroborated in supplemental experiments where we showed significant reductions in *C*. *muridarum*-induced IFN-β synthesis late during infection in gene-knockdown studies using IRF7-specific si-RNA. As also suggested by the IFN-β neutralization results, disrupting IRF7 gene expression early during infection had little impact on IFN-β synthesis, which implies that IRF7 is not involved early in this immune response. Collectively, our findings in OE cells corroborate the results shown in peritoneal macrophages by Pratner *et al* [28], as we demonstrate an early role for IRF3 and a late role for IRF7 during the *Chlamydia*-induced synthesis of IFN-β. However, the early IFN-β response to *Chlamydia* infection in OE cells is largely TLR3-dependent, which differs from the findings in peritoneal macrophages and implicates divergent pathways to IFN-β synthesis that converge and activate IRF3 in these two cell-types. The differential utilization of distinct IRF proteins during *C*. *muridarum* infection of OE cells likely lead to an ordered induction of IFN-β synthesis, resulting in tight control of these cytokines through a positive feedback mechanism [55].

We investigated the role of NF-κB in the *Chlamydia*-induced synthesis of IFN-β in OE cells, and we showed that inhibiting NF-κB activity with JSH-23 appeared to have only a minor effect on total IFN-β synthesized, but only when used early during infection. We routinely saw measurable decreases in overall IFN-β synthesis when JSH-23 was added at the 4hr PI time-point; however, the amount of decrease in the overall IFN-β secreted never quite reached statistical significance in our analyses. Interestingly, JSH-23 was much more effective in disrupting IFN-β synthesis in Bm1.11 cells that were transfected with poly-IC (*see Fig. 6C*). We previously reported that Bm1.11 cells did not synthesize IFN-β when poly-IC was added directly to the media, despite the robust expression of TLR3 in these cells [5]. The reason for the lack of response to extracellular poly-IC is poorly understood; however, we postulated that it may have been due to poly-IC’s inability to effectively enter into the BM1.11 cell endosome, due to poorly functioning micropinocytic pathways in the OE cells when compared to other cell types [56]. We found that transfecting the cells with poly-IC induced a very strong IFN-β response; however, the response was largely TRIF-independent [5]. The TRIF-independent induction of IFN-β by transfected poly-IC implicates other cytoplasmic receptors in OE cells that trigger type-1 IFN synthesis in response to double-stranded RNA (ds-RNA) such as RIG-I and MDA5 pathways [57–60]. Because both NF-κB and IRF3 play prominent roles in the synthesis of type-1 IFN in both the RIG-I and the MDA5 signaling pathway, it is conceivable that inhibitors of NF-κB and IRF3 would also disrupt signaling through these pathways. The relative contributions for both NF-κB and IRF3 might differ in RIG-I and MDA5 pathways when compared to TLR3 signaling pathways, which could possibly explain why inhibiting the function of NF-κB in these pathways has a much more dramatic effect on IFN-β synthesis in the poly-IC transfected OE cells.

In contrast to what we observed regarding the *Chlamydia*-induced IFN-β response, control reactions measuring IL-6 production in OE cells revealed that there were significant reductions in *Chlamydia*-induced synthesis of IL-6 throughout the course of infection in the JSH-23 treated cells. Collectively, our data implicate a much more impactful role for NF-κB in the synthesis of IL-6 during *Chlamydia* infection of OE cells, and that NF-κB pathways play a lessor role in the early infection induction of IFN-β. The significance of the relatively minor role for NF-κB in the TLR3-dependent synthesis of IFN-β is unclear; though, it is possible that it reflects the temporal relationship of when NF-κB and the IRF3 heterodimer are assembled into the IFN-β enhanceosome complex [61]. Investigations into assembly of the individual proteins into the human IFN-β enhanceosome complex suggest that NF-κB is assembled into the complex by binding to the positive regulatory domain II (PRDII) binding site of the IFN-β enhancer element, prior to the incorporation of the IRF3 heterodimer to the PRDI and III binding sites [62]. We have not examined the roles of IRF3 and NF-κB prior to the 4hr time point to ascertain whether NF-κB plays a more impactful role very early during *Chlamydia* infection of the OE cells. Future studies are necessary to address the possibility that NF-κB plays a more significant role in optimal IFN-β synthesis during *Chlamydia* infection at time points earlier than the 4hr PI, and to address the hypothesis that NF-κB is more heavily recruited to the enhanceosome complex prior to the 4hr time point.

It is well known that TLR3 recognizes ds-RNA during viral infection to induce type-I interferon synthesis in a variety of different cell types [32,57]. Others have identified an inosine-containing single-stranded RNA PAMP that induces TLR3-dependent responses to respiratory syncytial virus (RSV) infections [63]. Although we have demonstrated a TLR3-dependent IFN-β response to *C*. *muridarum* in the OE cells [6,14], the actual chlamydial PAMP that stimulates the TLR3-dependent responses is currently unknown. Because there is no known ds-RNA moiety associated with the chlamydial structure, we hypothesized that it was either an unconventional TLR3 PAMP presented during *C*. *muridarum* infection (such as bacterial DNA or RNA), or that *C*. *muridarum* induced a cellular ds-RNA during infection that served as a TLR3 PAMP. Our data demonstrate that inhibition of bacterial DNA replication at early-to-mid infection had the most dramatic impact on overall *Chlamydia*-induced IFN-β synthesis in OE cells, while inhibition of bacterial transcription had virtually no impact on cellular IFN-β synthesis. The significance of this finding is unclear; however, it would support the hypothesis that *C*. *muridarum* DNA replication produces a PAMP that augments TLR3-dependent IFN-β synthesis in these cells. Our data underscores the possibility of chlamydial DNA being that unconventional TLR3 PAMP that is presented during the course of *C*. *muridarum* infection in the OE cells, or that the chlamydial DNA enhances overall IFN-β synthesis through other pathways the involve cellular DNA sensors that can signal type-1 IFN responses via STING as an alternative mechanism [42,64]. Studies are underway that will further address the putative role of *Chlamydia* DNA as a possible TLR3 PAMP in OE cells.

## Supporting Information

S1 Fig *Chlamydia* Conditioned media induces transcription in uninfected OE cells. Supernatants were removed from *C*. *muridarum* infected Bm1.11 OE cells that were incubated in presence or absence of cycloheximide for the 4 hour intervals indicated. The conditioned supernatants were added to uninfected Bm1.11 cells for 4 h before monolayers were harvested for RNA isolation to examine effect on transcription rates of: **(A)** components of type-1 IFN signaling pathways, **(B)** various inflammatory mediators, and **(C)** TLRs known to be expressed and functional in Bm1.11 OE cells. *The results shown are representative of three independent experiments*. *Cyclo = 1*μ*M cycloheximide*.
(TIF)

S2 Fig Effect of non-viable *C*. *muridarum* on IFNAR1 gene transcription. Bm1.11 cells were either mock-infected or infected with 10 IFU/ cell *C*. *muridarum* that has been heat-inactivated at 56°C for 30 minutes (see Materials and Methods). Gene expression levels of IFNAR1 was measured by RT-qPCR after total cell mRNA was harvested at 24 h post-infection. *The results shown are representative of three independent experiments; MoPn (HK) = heat killed C*. *muridarum*.
(TIF)

S3 Fig IRF7 has a role in the *Chlamydia*-induced synthesis of IFN-β at late times during infection. Bm1.1 cells were infected with 10 IFU/ cell *C*. *muridarum* 24 h after transfection with either si-RNA specific for IRF7 *(si-IRF7)*, scrambled control si-RNA *(si-SCR)*, or lipofectamine-only control *(MoPn)*. **(A)** RT-qPCR results showing the gene expression levels of IRF7 after total cell mRNA was harvested at either early (12h) or late (24h) times post infection. **(B)** Western blot analysis showing IRF7 protein expression at 12 and 24 h post-infection. **(C)** *Chlamydia*-induced IFN-β levels in the supernatants of each group were determined by ELISA at 12 and 24 h post-infection. *The results shown are representative of three independent experiments*. *Statistical significance was determined by comparing the treatment conditions of si-IRF7–transfected Bm1*.*11 cells with Bm1*.*11 cells transfected with lipofectamine-only*. ** = *p < 0*.*01*.
(TIF)

S4 Fig Role of IRF7 in TLR9-dependent IFN-β synthesis. **(A)** ELISA showing IFN-β levels detected in supernatants from RAW 264.7 cells infected with 10 IFU/ cell *C*. *muridarum*, *or* stimulated with either the TLR9 agonist ODN1826 or ODN1826 control. **(B)** ELISA showing IFN-β synthesis in RAW 264.7 cells that were stimulated with ODN1826, or stimulated with ODN1826 24 h after transfection with either si-RNA specific for IRF7 *(si-IRF7)* or scrambled control si-RNA *(si-SCR)*. **(C)** IFN-β was measured in RAW 264.7 cells that were stimulated with ODN1826 in the absence or presence of increasing concentrations of the IRF3 inhibitor BX-795. *The results shown are representative of three independent experiments*. *Statistical significance was determined by comparing the specified condition versus RAW264*.7 cells treated with ODN1826; * = p < 0.*05; NS = not statistically significant*.
(TIF)

S5 Fig Inhibition of bacterial DNA replication and bacterial transcription in *Chlamydia*-infected OE cells. Bm1.1 cells were infected with 10 IFU/ cell *C*. *muridarum* and cells were incubated in the presence of increasing concentrations of either rifampicin or ofloxacin starting at 2h PI. The medium was replaced with antibiotic-free medium at 18h PI, cells were harvested at 30h PI for analysis of chlamydial gene transcription and DNA replication. **(A)** Quantitative PCR using primers specific for *omp1* to measure chlamydial DNA replication at the 30 h time-point. **(B)** RT-qPCR showing transcription of the *Chlamydia*-specific 16S rRNA versus the cellular β-Actin gene. *The results shown are representative of three independent experiments*.
(TIF)
