# Supplementary figures and images for: Analyses of the Pathways Involved in Early- and Late-Phase Induction of IFN-Beta during C. muridarum Infection of Oviduct Epithelial Cells
Source: PLoS One. 2015 Mar 23;10(3):e0119235. doi: 10.1371/journal.pone.0119235 (PMC4370658; doi:10.1371/journal.pone.0119235)

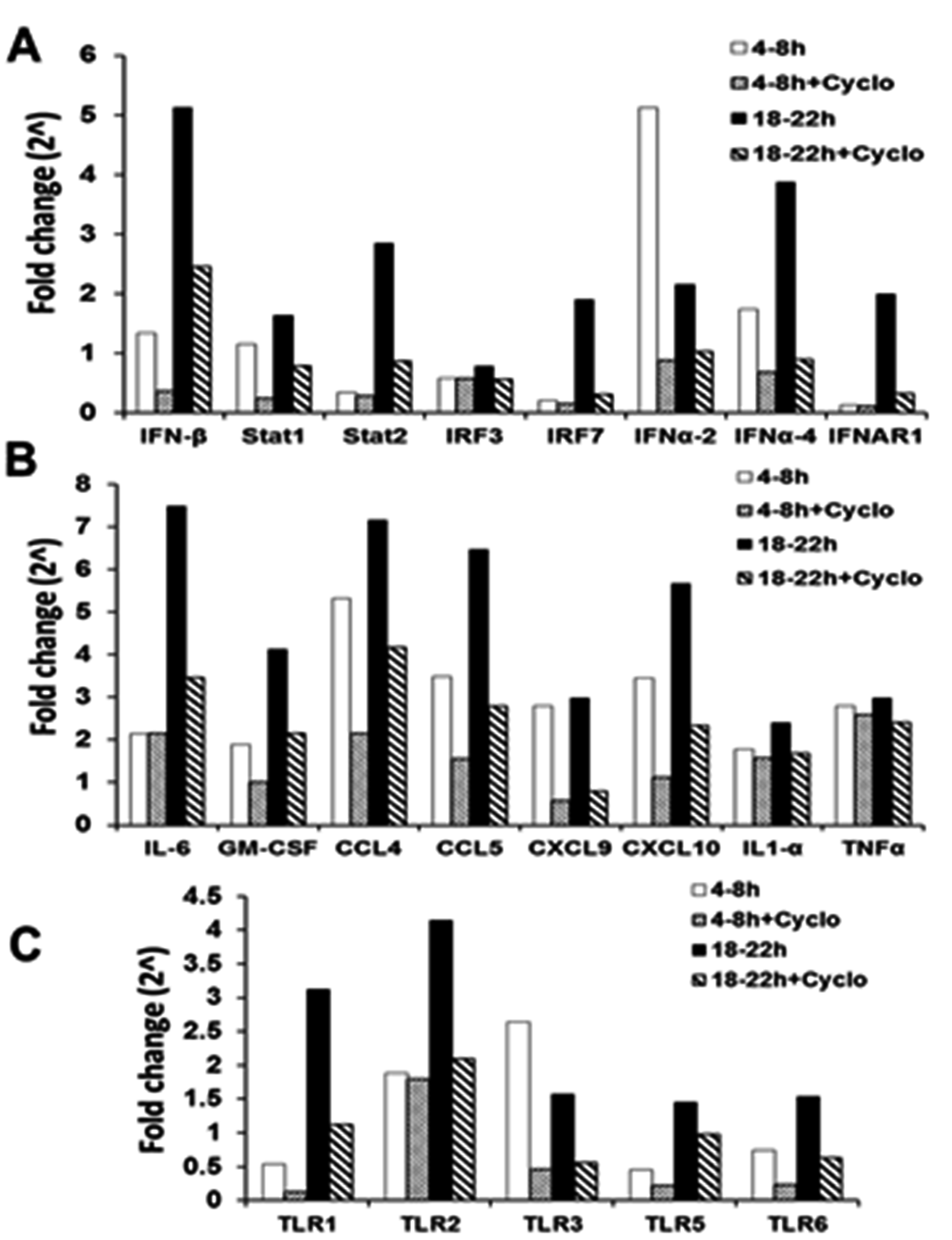

Supplement: S1 Fig — Supernatants were removed from C. muridarum infected Bm1.11 OE cells that were incubated in presence or absence of cycloheximide for the 4 hour intervals indicated. The conditioned supernatants were added to uninfected Bm1.11 cells for 4 h before monolayers were harvested for RNA isolation to examine effect on transcription rates of: (A) components of type-1 IFN signaling pathways, (B) various inflammatory mediators, and (C) TLRs known to be expressed and functional in Bm1.11 OE cells. The results shown are representative of three independent experiments. Cyclo = 1μM cycloheximide. (TIF) [file pone.0119235.s001.tif]

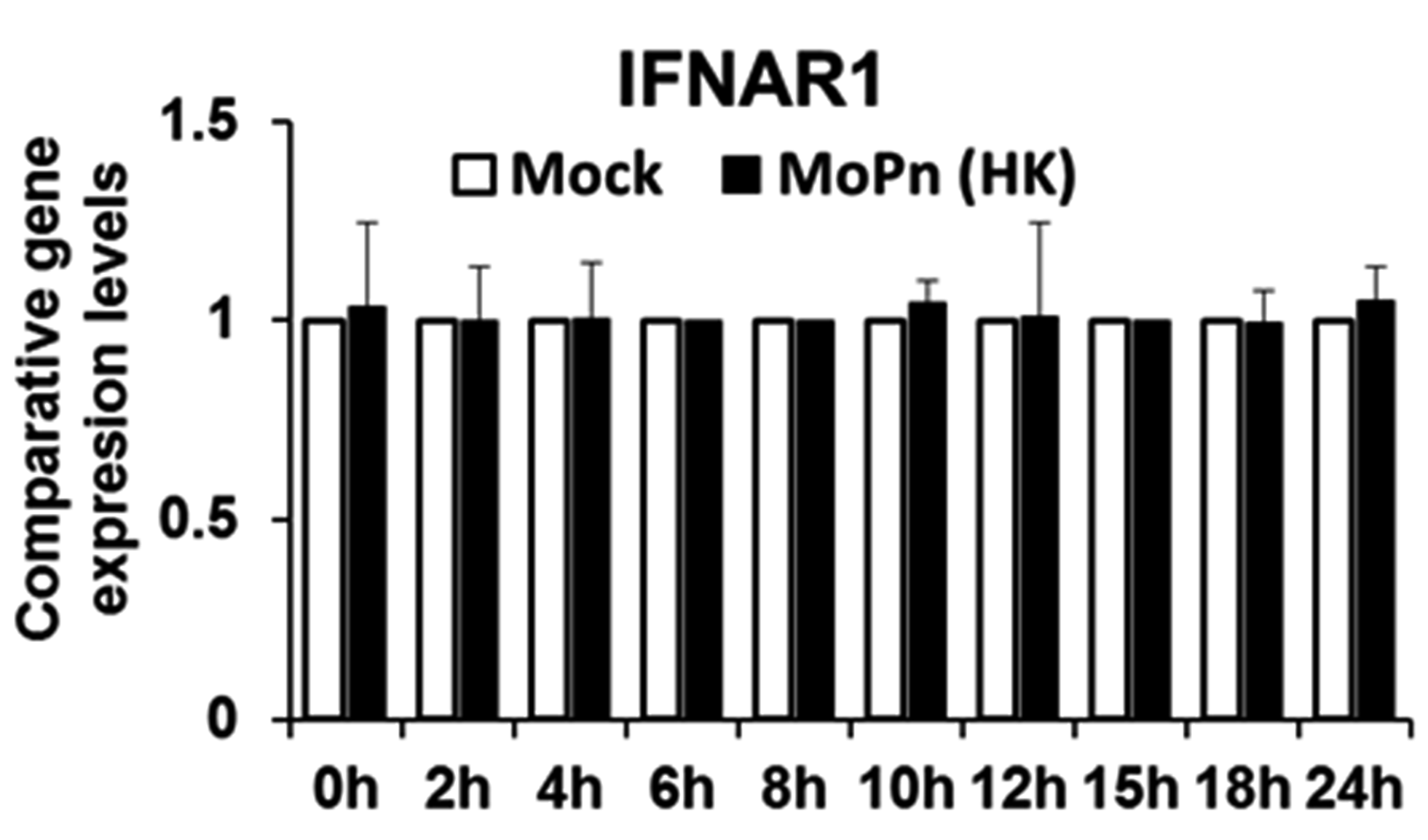

Supplement: S2 Fig — Bm1.11 cells were either mock-infected or infected with 10 IFU/ cell C. muridarum that has been heat-inactivated at 56°C for 30 minutes (see Materials and Methods). Gene expression levels of IFNAR1 was measured by RT-qPCR after total cell mRNA was harvested at 24 h post-infection. The results shown are representative of three independent experiments; MoPn (HK) = heat killed C. muridarum. (TIF) [file pone.0119235.s002.tif]

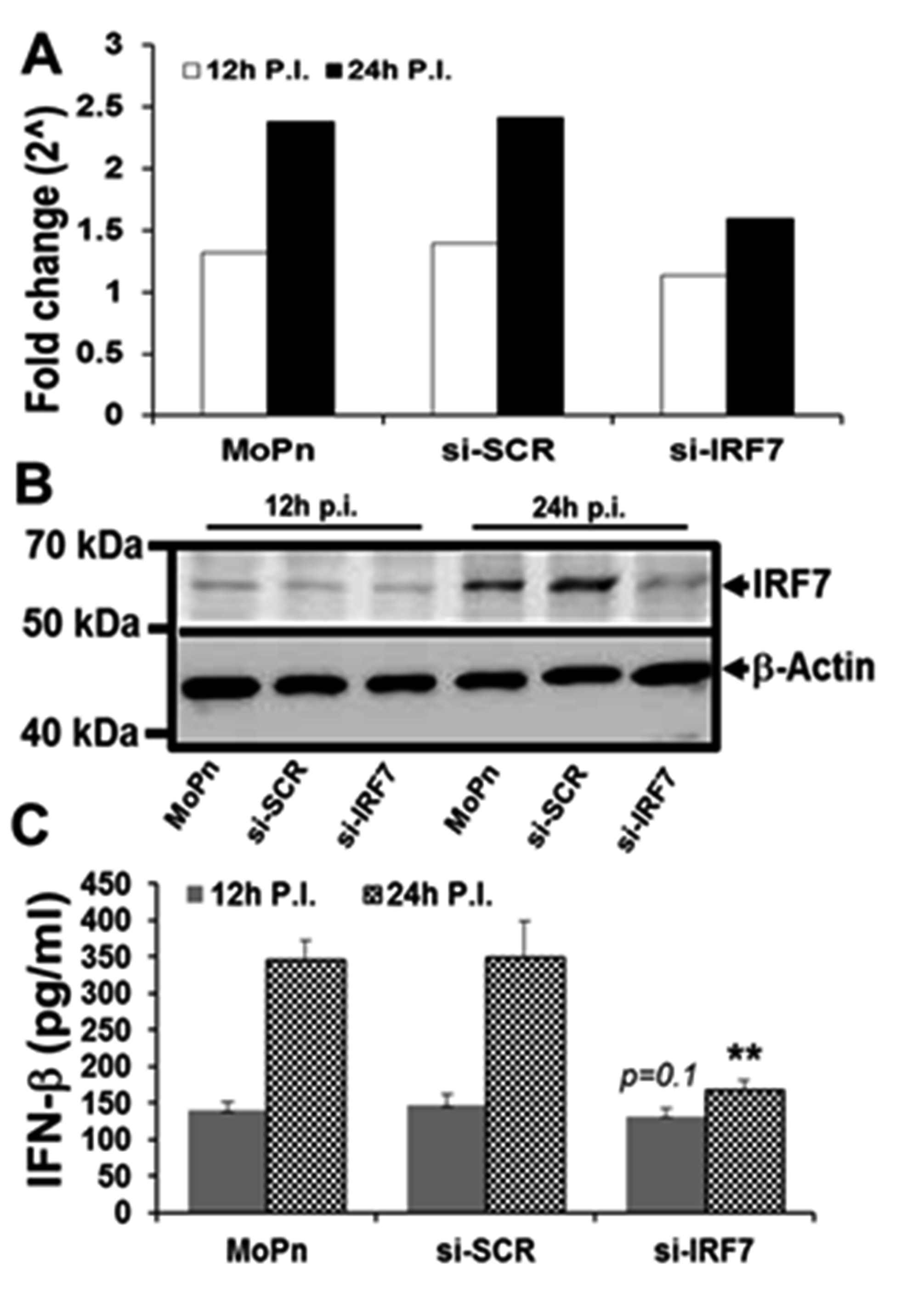

Supplement: S3 Fig — Bm1.1 cells were infected with 10 IFU/ cell C. muridarum 24 h after transfection with either si-RNA specific for IRF7 (si-IRF7), scrambled control si-RNA (si-SCR), or lipofectamine-only control (MoPn). (A) RT-qPCR results showing the gene expression levels of IRF7 after total cell mRNA was harvested at either early (12h) or late (24h) times post infection. (B) Western blot analysis showing IRF7 protein expression at 12 and 24 h post-infection. (C) Chlamydia-induced IFN-β levels in the supernatants of each group were determined by ELISA at 12 and 24 h post-infection. The results shown are representative of three independent experiments. Statistical significance was determined by comparing the treatment conditions of si-IRF7–transfected Bm1.11 cells with Bm1.11 cells transfected with lipofectamine-only. ** = p < 0.01. (TIF) [file pone.0119235.s003.tif]

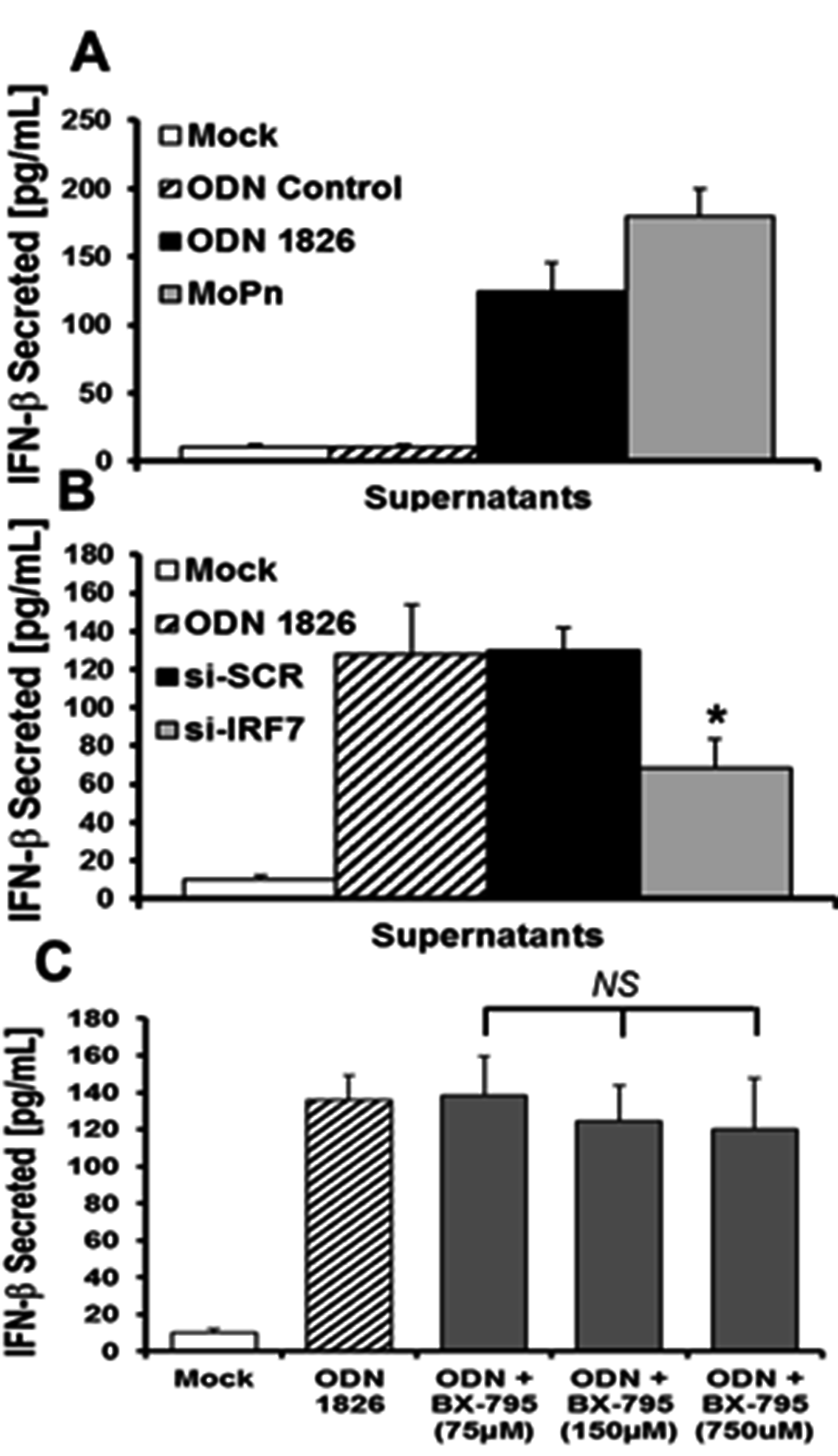

Supplement: S4 Fig — (A) ELISA showing IFN-β levels detected in supernatants from RAW 264.7 cells infected with 10 IFU/ cell C. muridarum, or stimulated with either the TLR9 agonist ODN1826 or ODN1826 control. (B) ELISA showing IFN-β synthesis in RAW 264.7 cells that were stimulated with ODN1826, or stimulated with ODN1826 24 h after transfection with either si-RNA specific for IRF7 (si-IRF7) or scrambled control si-RNA (si-SCR). (C) IFN-β was measured in RAW 264.7 cells that were stimulated with ODN1826 in the absence or presence of increasing concentrations of the IRF3 inhibitor BX-795. The results shown are representative of three independent experiments. Statistical significance was determined by comparing the specified condition versus RAW264.7 cells treated with ODN1826; * = p < 0.05; NS = not statistically significant. (TIF) [file pone.0119235.s004.tif]

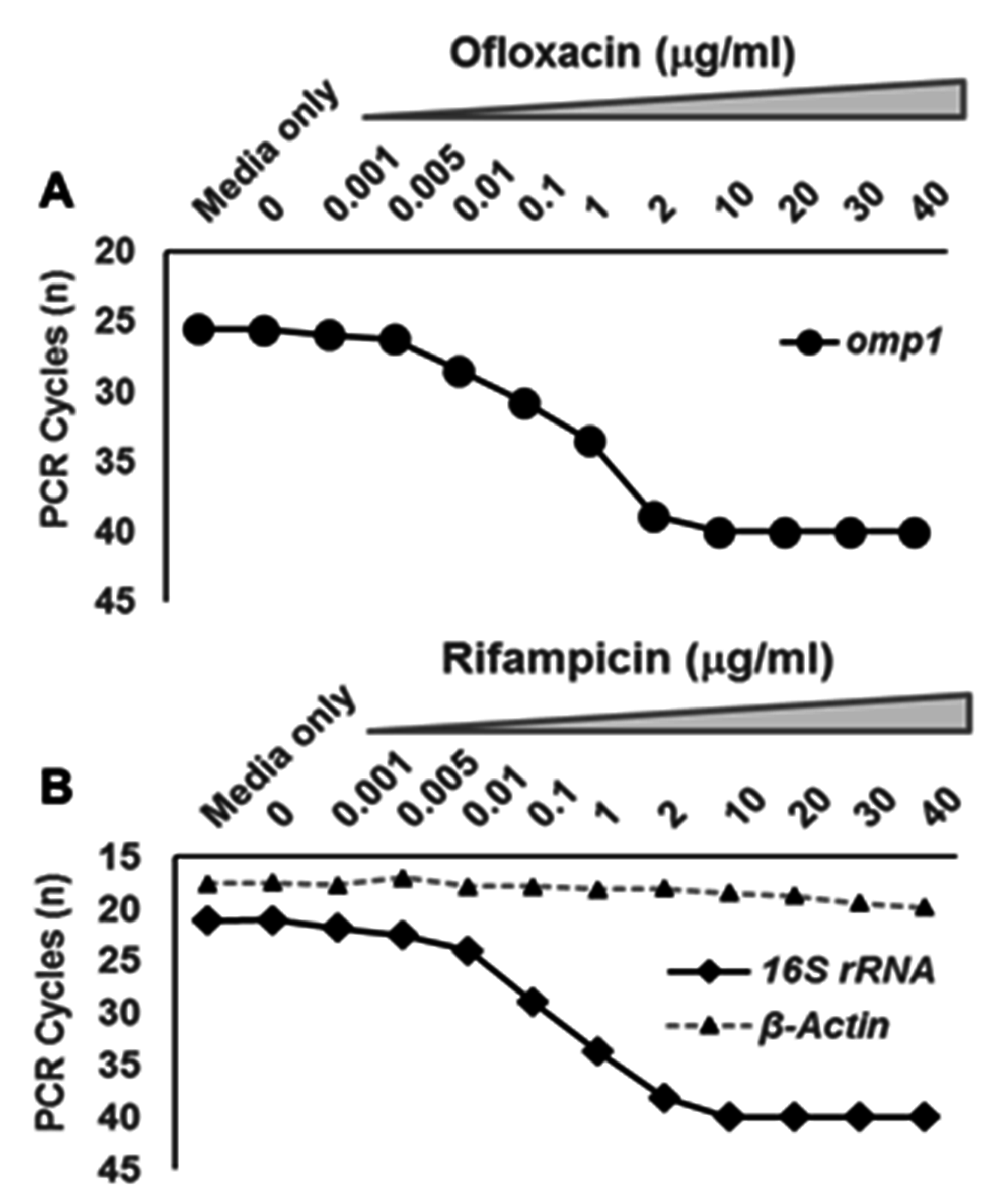

Supplement: S5 Fig — Bm1.1 cells were infected with 10 IFU/ cell C. muridarum and cells were incubated in the presence of increasing concentrations of either rifampicin or ofloxacin starting at 2h PI. The medium was replaced with antibiotic-free medium at 18h PI, cells were harvested at 30h PI for analysis of chlamydial gene transcription and DNA replication. (A) Quantitative PCR using primers specific for omp1 to measure chlamydial DNA replication at the 30 h time-point. (B) RT-qPCR showing transcription of the Chlamydia-specific 16S rRNA versus the cellular β-Actin gene. The results shown are representative of three independent experiments. (TIF) [file pone.0119235.s005.tif]
